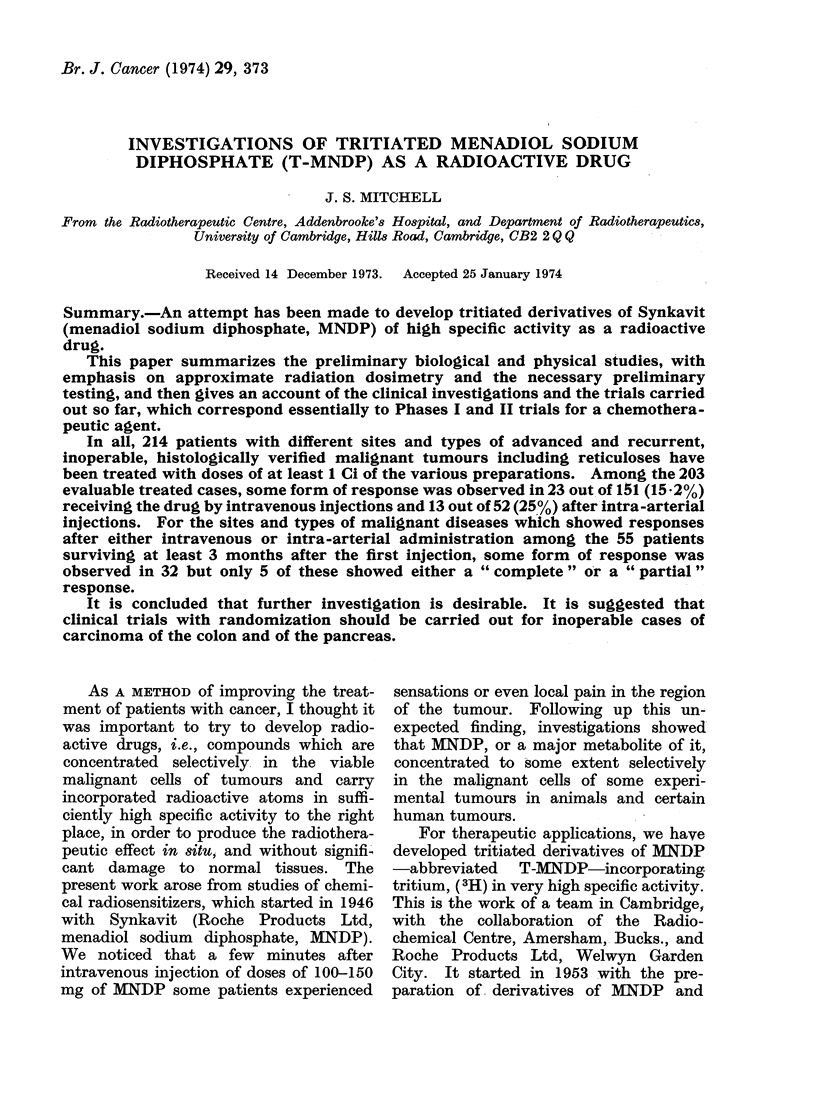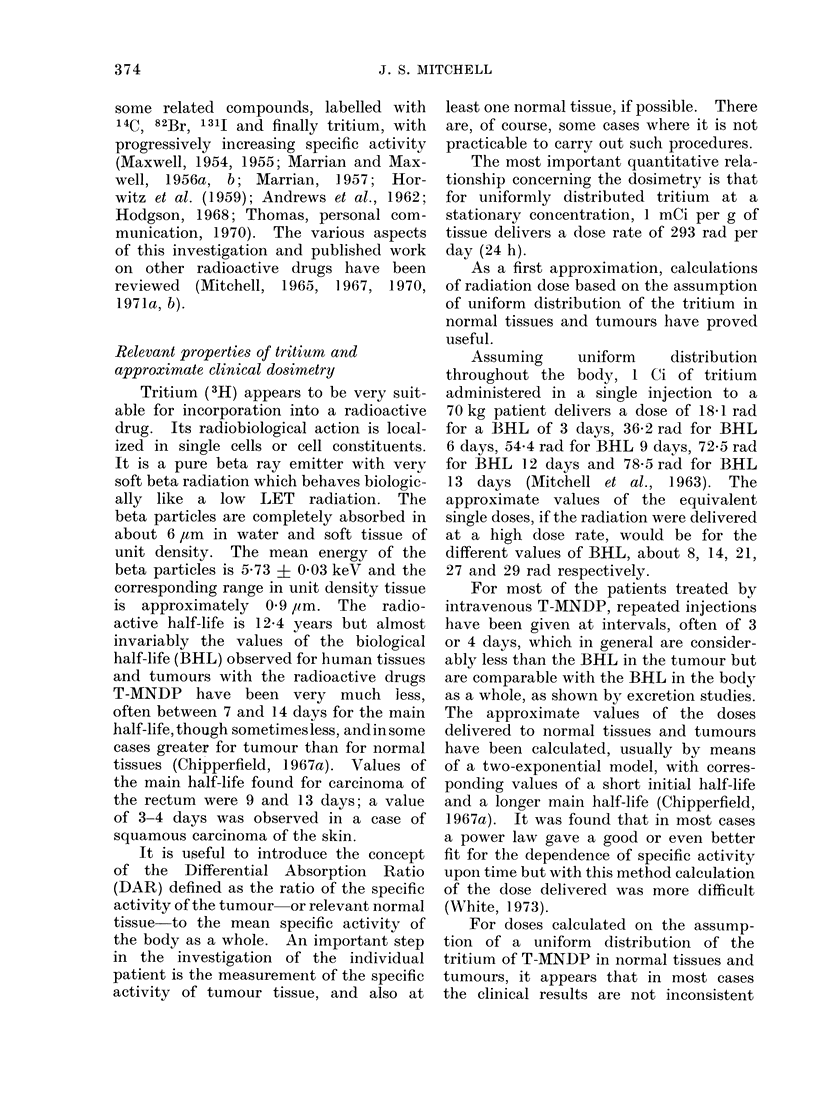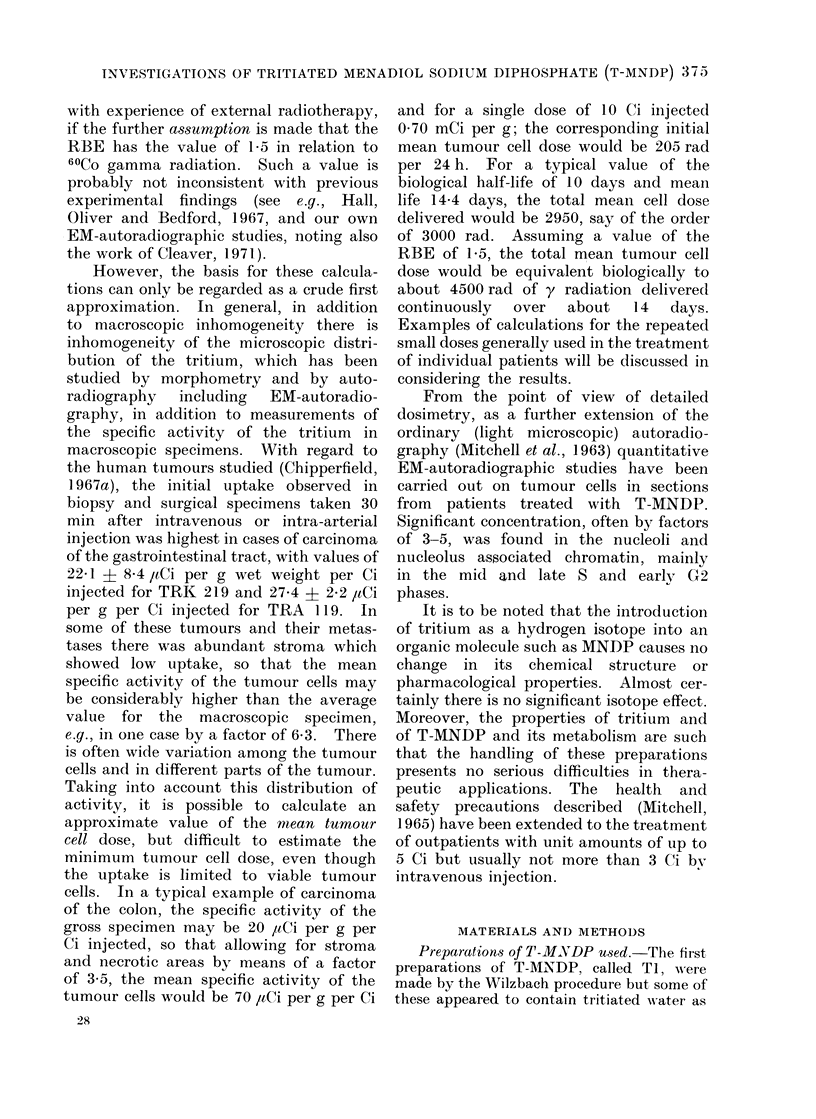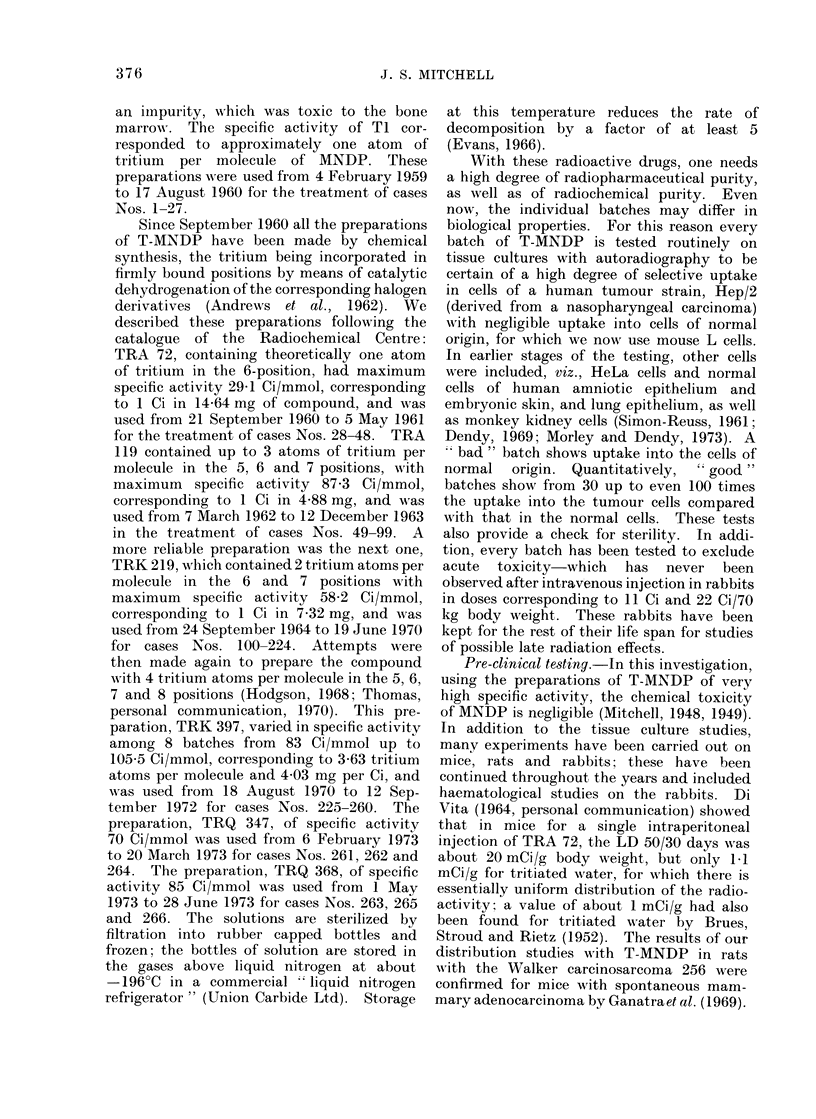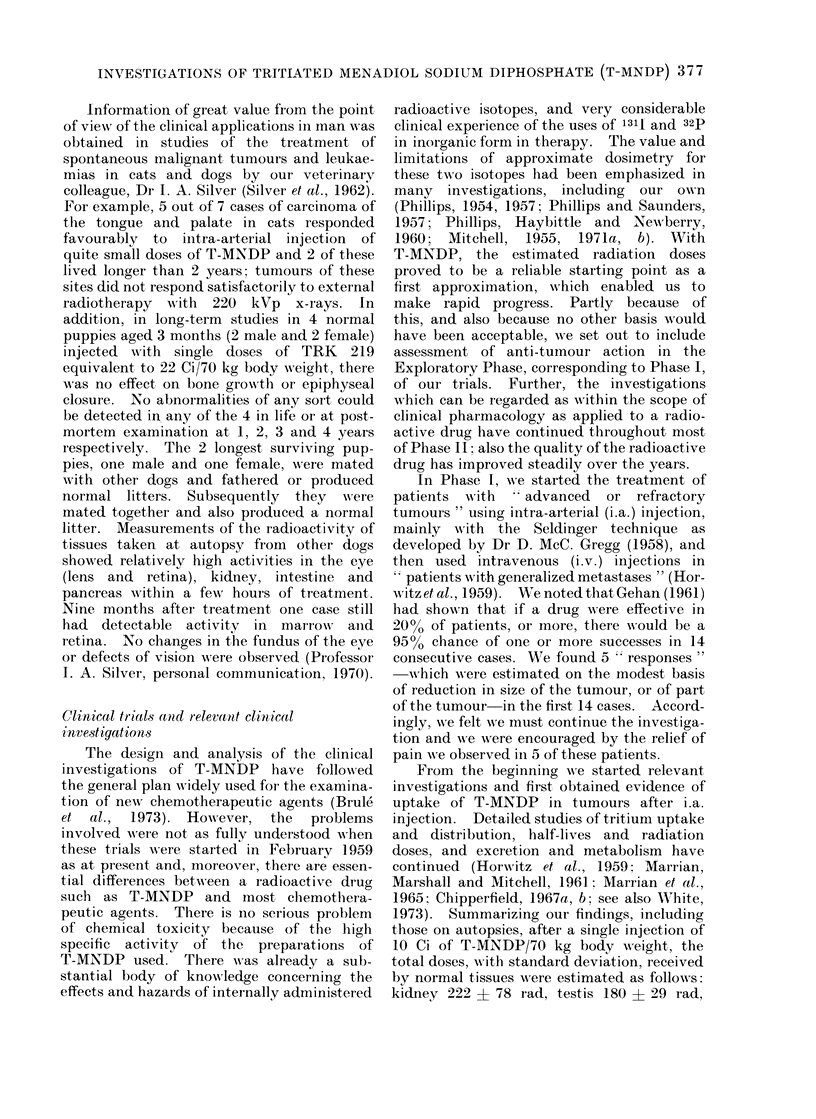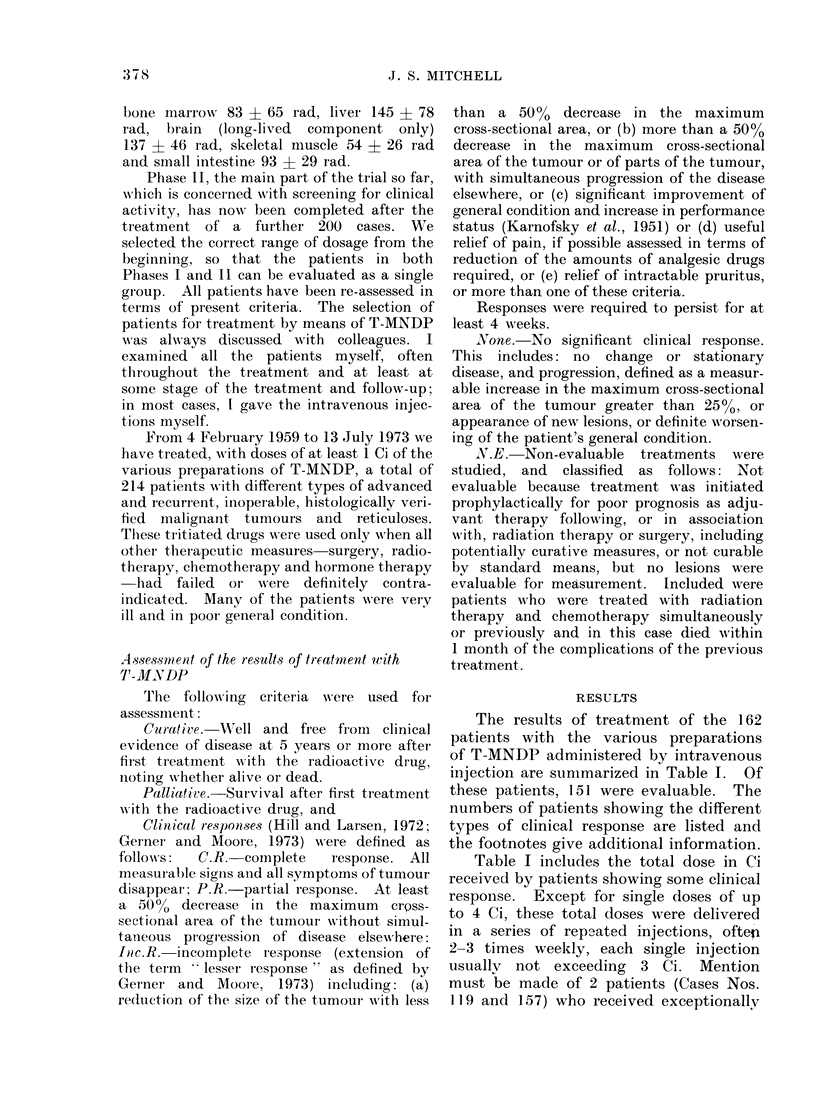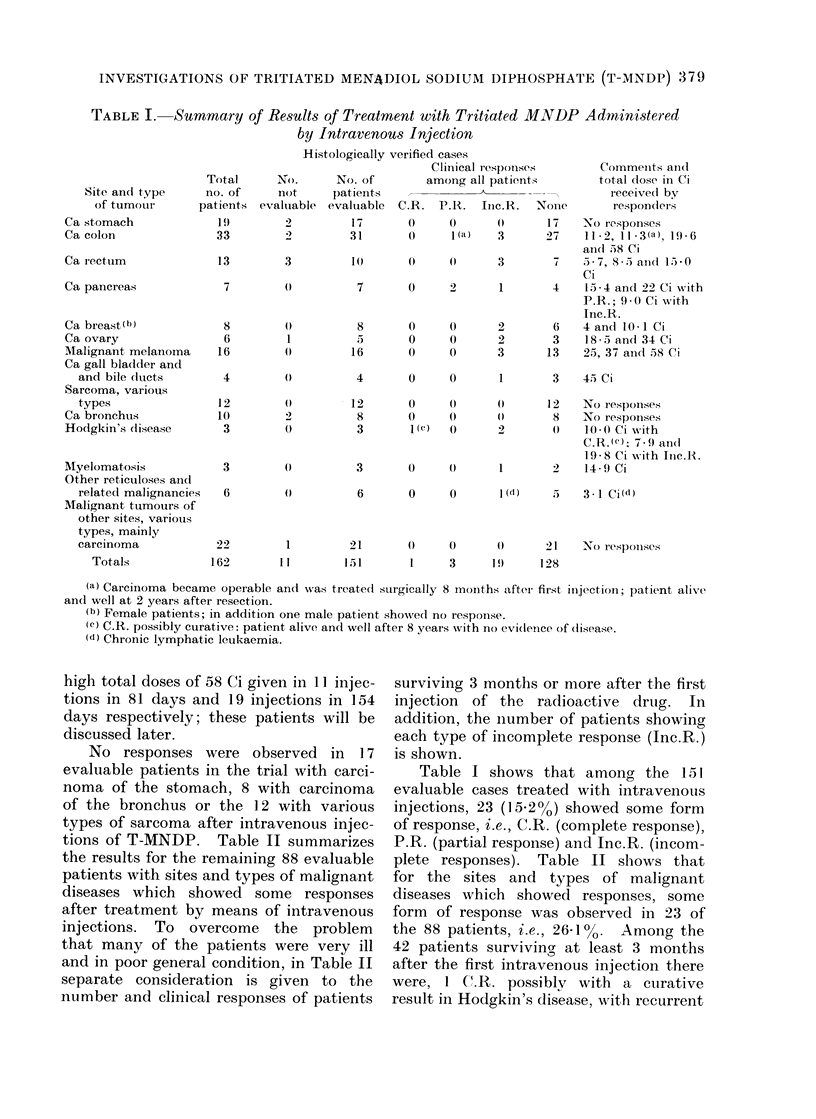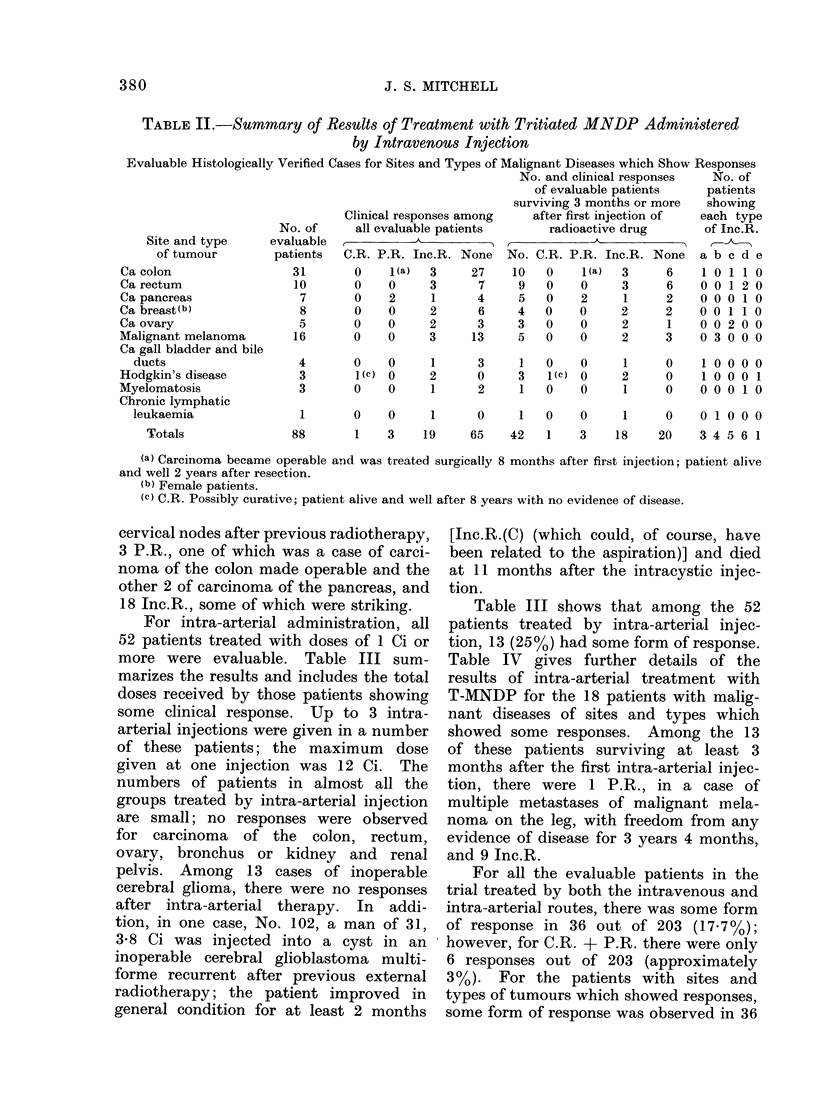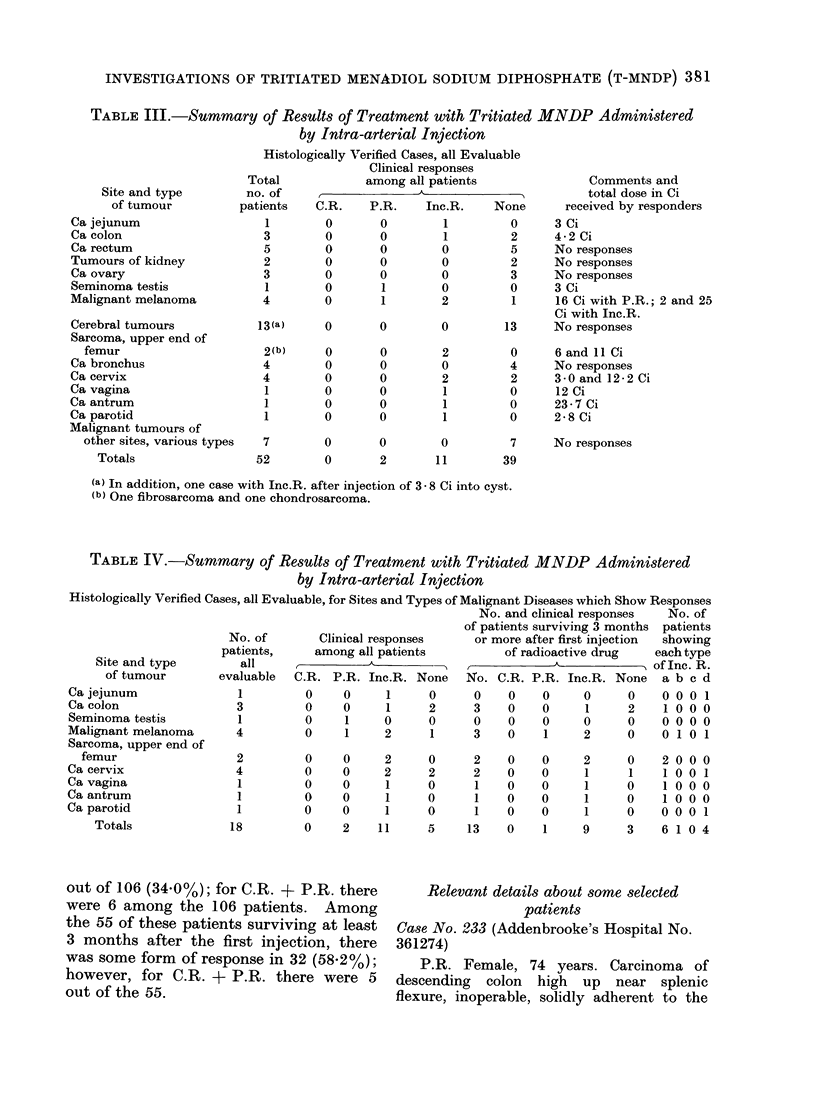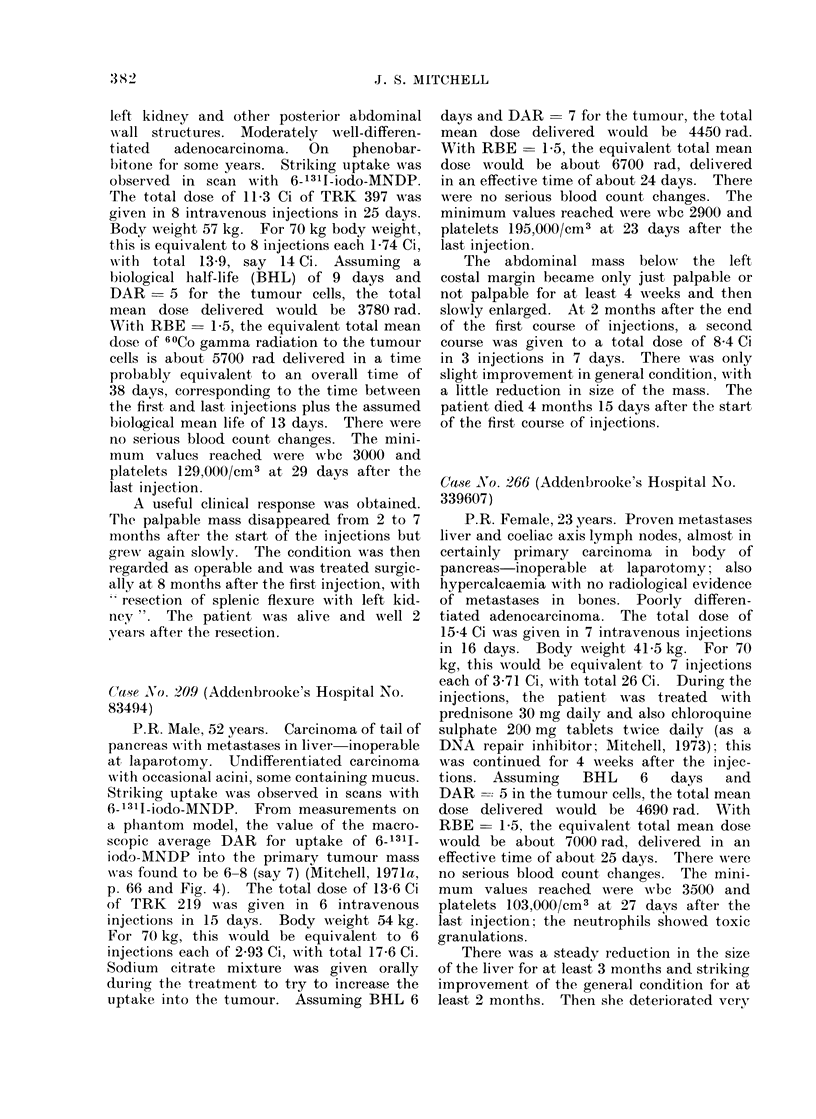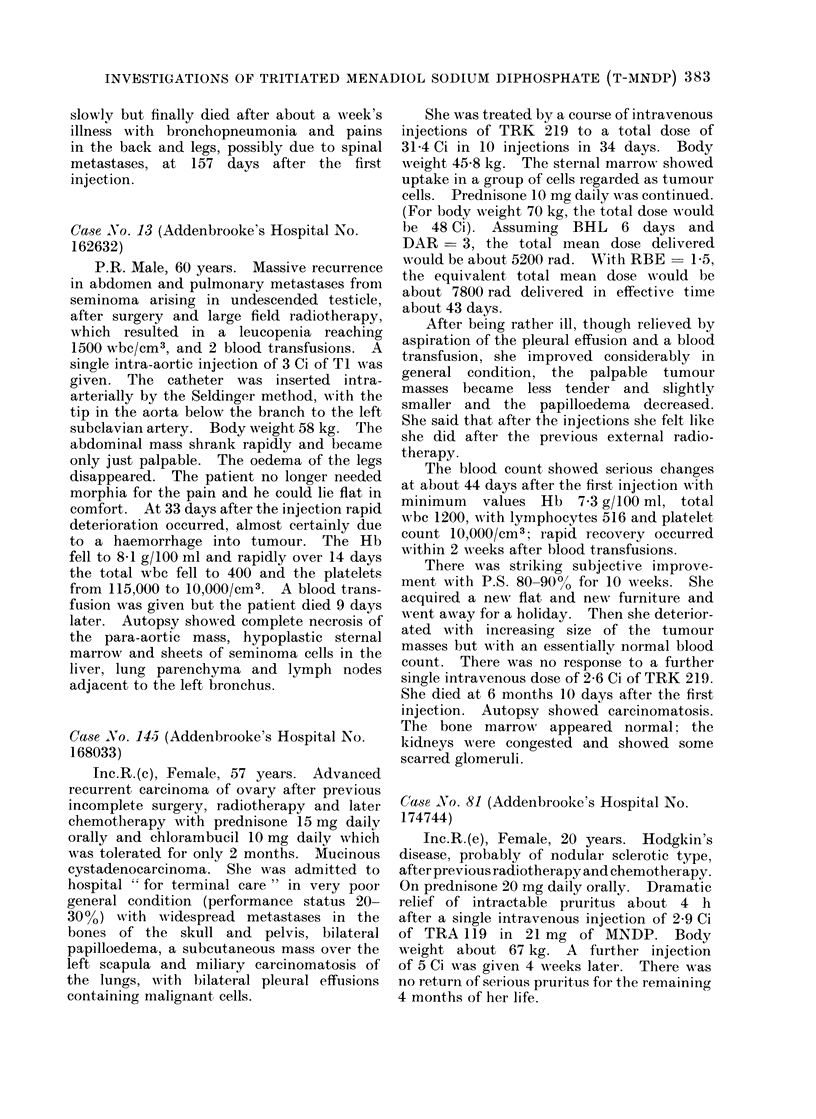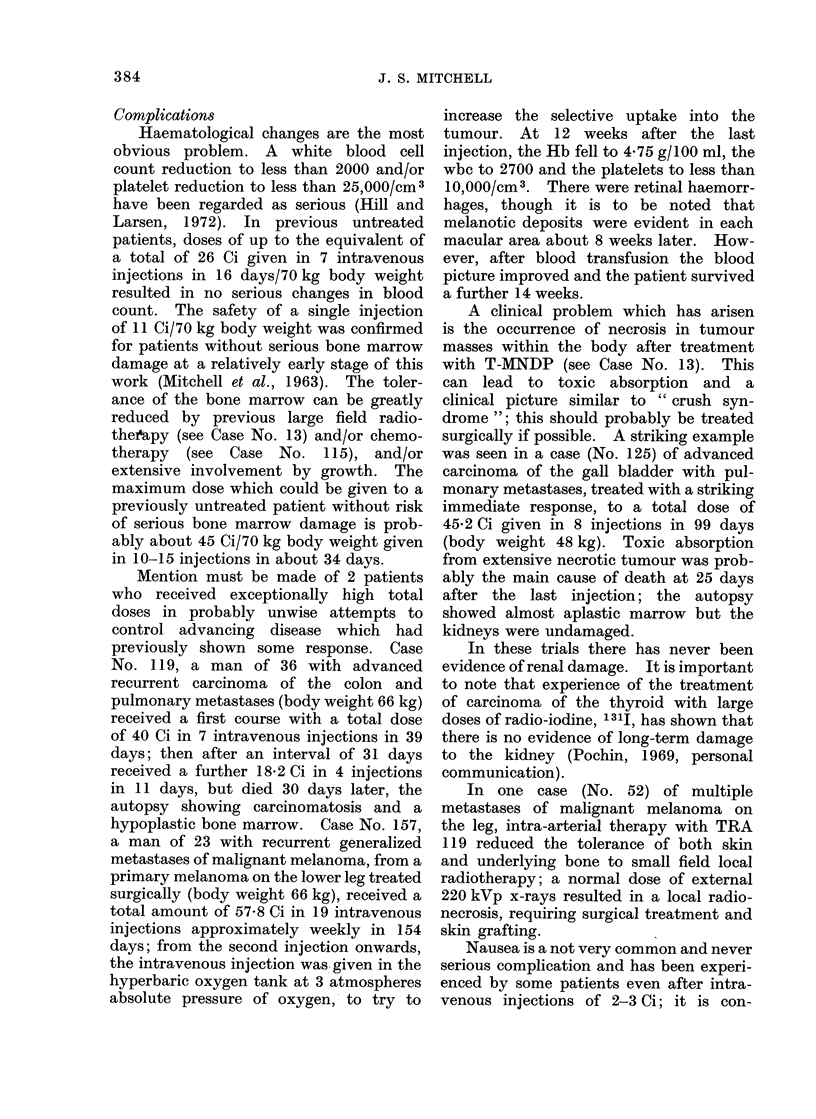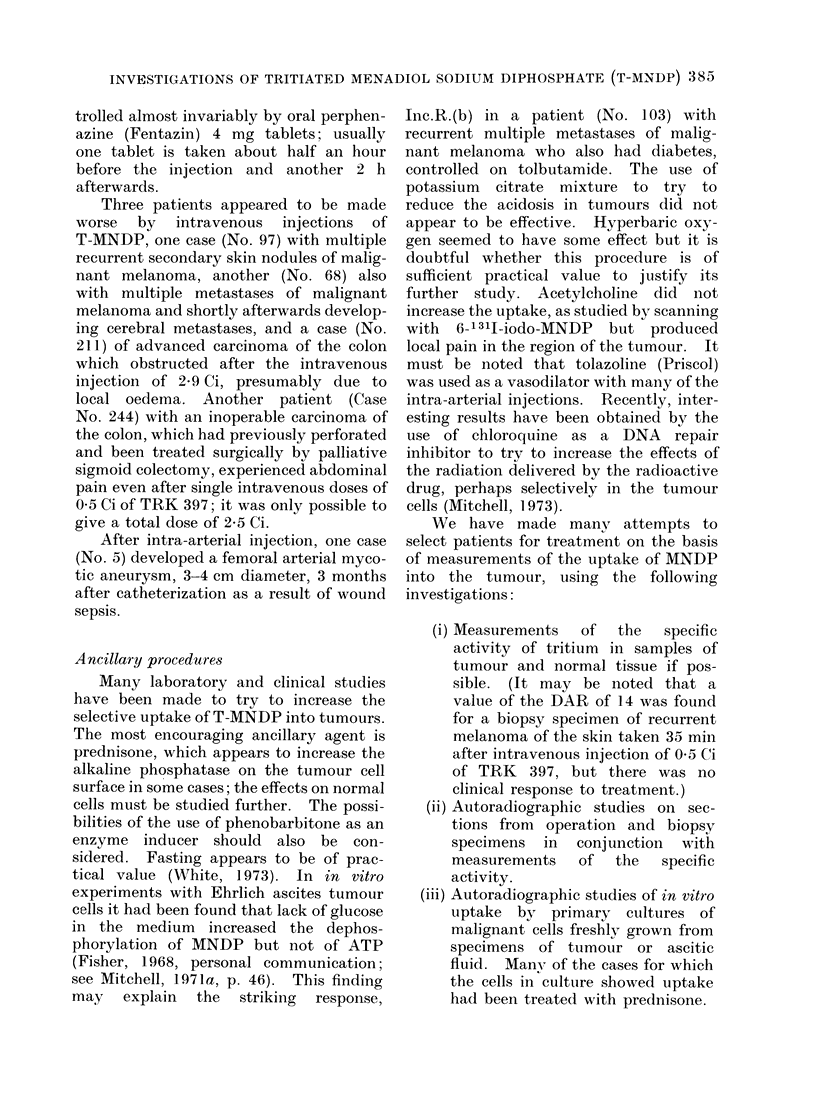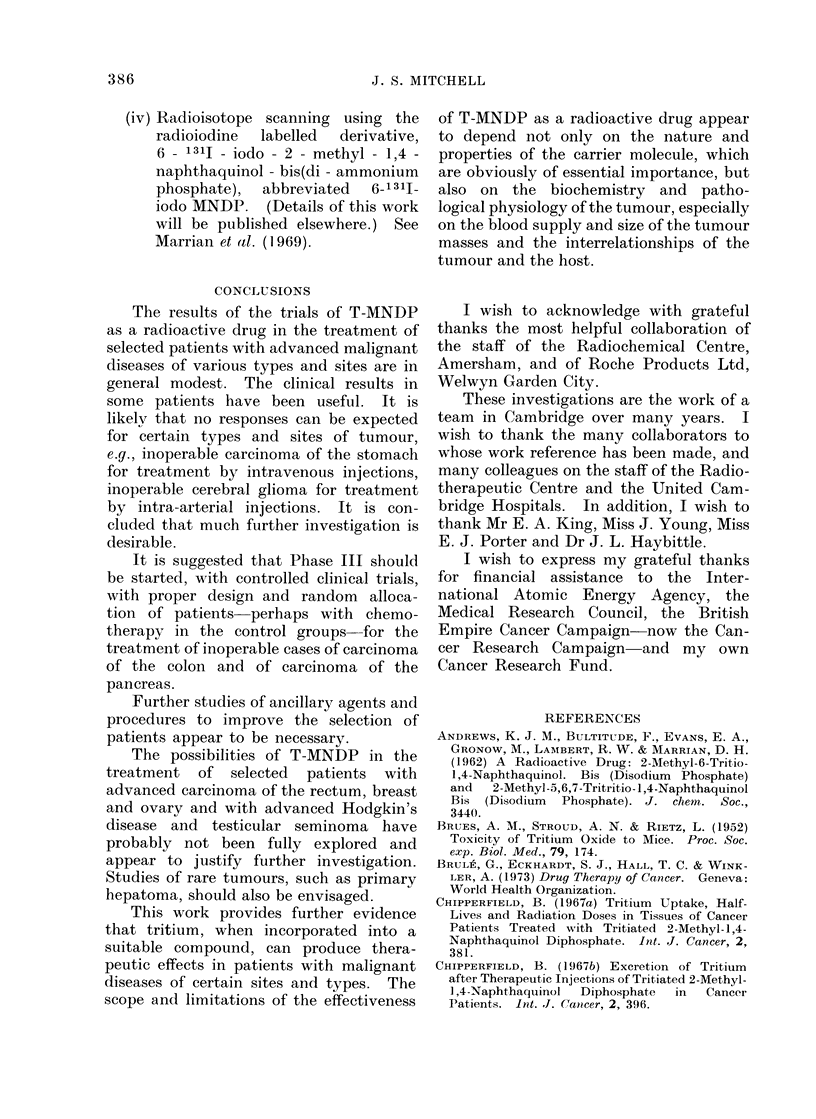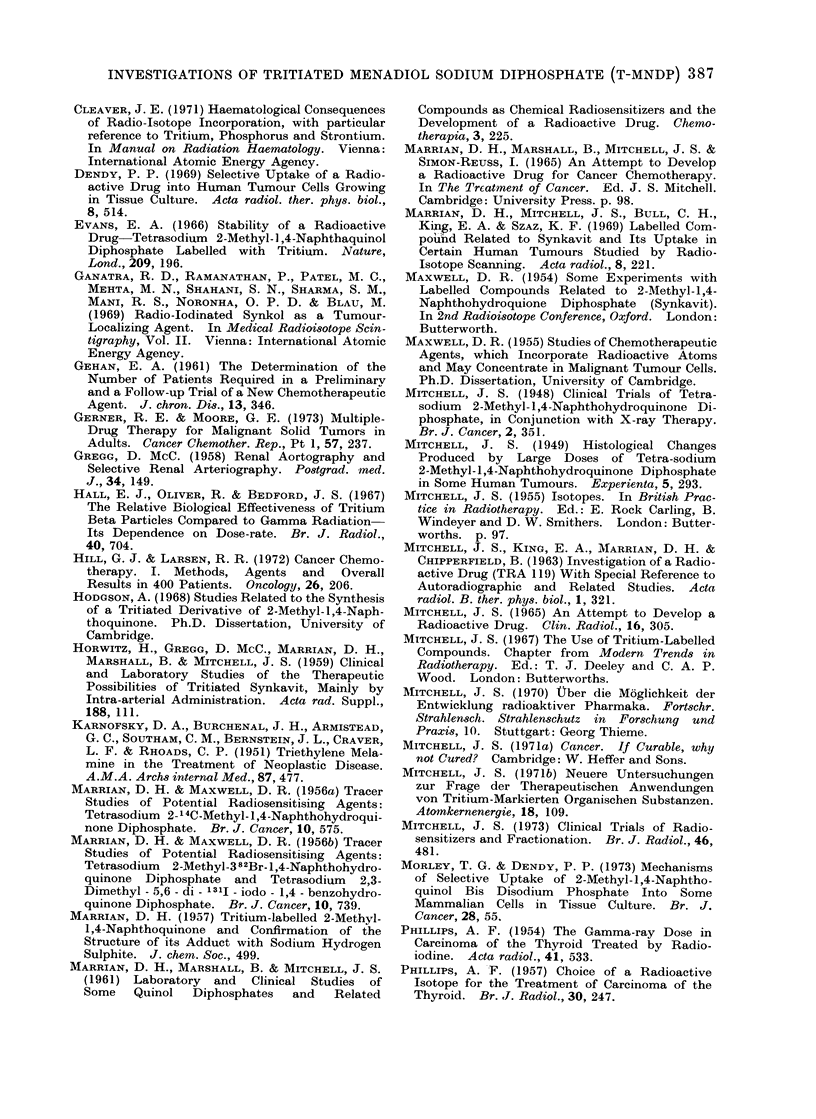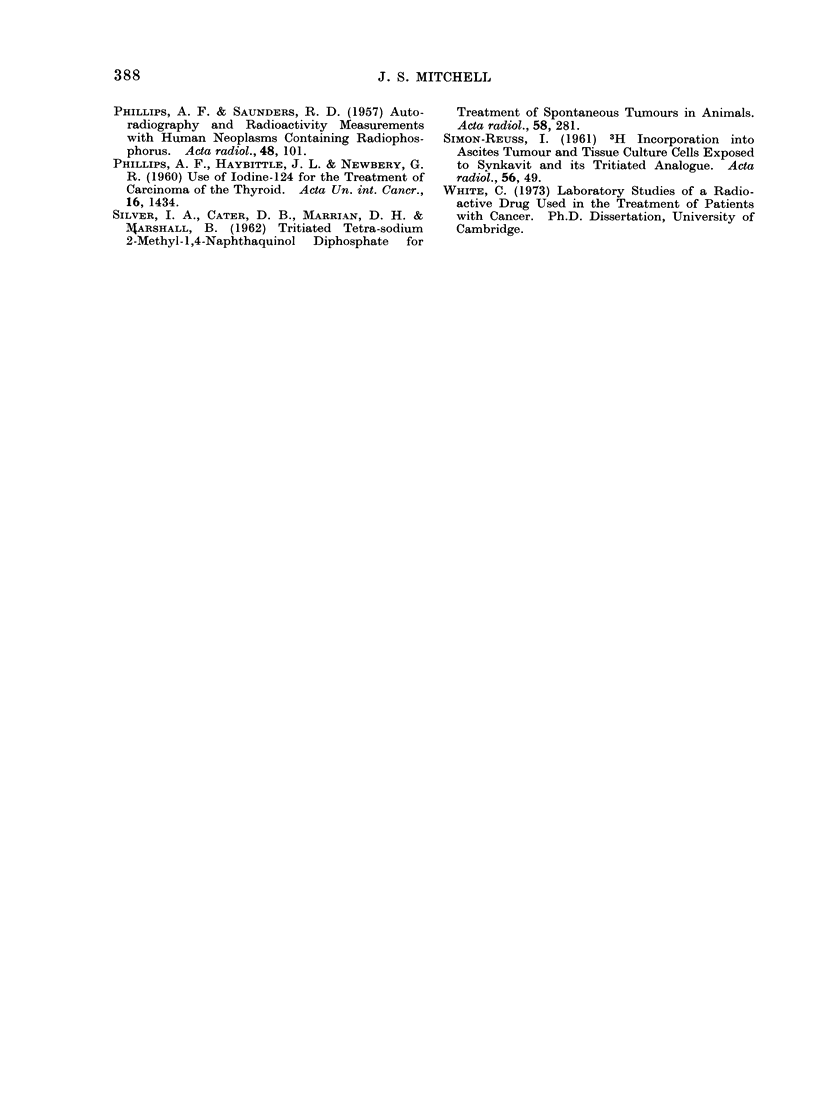# Investigations of Tritiated Menadiol Sodium Diphosphate (T-MNDP) as a Radioactive Drug

**DOI:** 10.1038/bjc.1974.85

**Published:** 1974-05

**Authors:** J. S. Mitchell

## Abstract

An attempt has been made to develop tritiated derivatives of Synkavit (menadiol sodium diphosphate, MNDP) of high specific activity as a radioactive drug.

This paper summarizes the preliminary biological and physical studies, with emphasis on approximate radiation dosimetry and the necessary preliminary testing, and then gives an account of the clinical investigations and the trials carried out so far, which correspond essentially to Phases I and II trials for a chemotherapeutic agent.

In all, 214 patients with different sites and types of advanced and recurrent, inoperable, histologically verified malignant tumours including reticuloses have been treated with doses of at least 1 Ci of the various preparations. Among the 203 evaluable treated cases, some form of response was observed in 23 out of 151 (15·2%) receiving the drug by intravenous injections and 13 out of 52 (25%) after intra-arterial injections. For the sites and types of malignant diseases which showed responses after either intravenous or intra-arterial administration among the 55 patients surviving at least 3 months after the first injection, some form of response was observed in 32 but only 5 of these showed either a “complete” or a “partial” response.

It is concluded that further investigation is desirable. It is suggested that clinical trials with randomization should be carried out for inoperable cases of carcinoma of the colon and of the pancreas.


Br. J. Cancer (1974) 29, 373

### INVESTIGATIONS OF TRITIATED MENADIOL SODIUM DIPHOSPHATE (T-MNDP) AS A RADIOACTIVE DRUG

J. S. MITCHELL

*From the Radiotherapeutic Centre, Addenbrooke's Hospital, and Department of Radiotherapeutics, University of Cambridge, Hills Road, Cambridge, CB2 2QQ*

Received 14 December 1973. Accepted 25 January 1974

**Summary.—An attempt has been made to develop tritiated derivatives of Synkavit (menadiol sodium diphosphate, MNDP) of high specific activity as a radioactive drug.**

**This paper summarizes the preliminary biological and physical studies, with emphasis on approximate radiation dosimetry and the necessary preliminary testing, and then gives an account of the clinical investigations and the trials carried out so far, which correspond essentially to Phases I and II trials for a chemotherapeutic agent.**

**In all, 214 patients with different sites and types of advanced and recurrent, inoperable, histologically verified malignant tumours including reticuloses have been treated with doses of at least 1 Ci of the various preparations. Among the 203 evaluable treated cases, some form of response was observed in 23 out of 151 (15·2%) receiving the drug by intravenous injections and 13 out of 52 (25%) after intra-arterial injections. For the sites and types of malignant diseases which showed responses after either intravenous or intra-arterial administration among the 55 patients surviving at least 3 months after the first injection, some form of response was observed in 32 but only 5 of these showed either a "complete" or a "partial" response.**

**It is concluded that further investigation is desirable. It is suggested that clinical trials with randomization should be carried out for inoperable cases of carcinoma of the colon and of the pancreas.**

As A METHOD of improving the treatment of patients with cancer, I thought it was important to try to develop radioactive drugs, *i.e.*, compounds which are concentrated selectively in the viable malignant cells of tumours and carry incorporated radioactive atoms in sufficiently high specific activity to the right place, in order to produce the radiotherapeutic effect *in situ*, and without significant damage to normal tissues. The present work arose from studies of chemical radiosensitizers, which started in 1946 with Synkavit (Roche Products Ltd, menadiol sodium diphosphate, MNDP). We noticed that a few minutes after intravenous injection of doses of 100–150 mg of MNDP some patients experienced sensations or even local pain in the region of the tumour. Following up this unexpected finding, investigations showed that MNDP, or a major metabolite of it, concentrated to some extent selectively in the malignant cells of some experimental tumours in animals and certain human tumours.

For therapeutic applications, we have developed tritiated derivatives of MNDP —abbreviated T-MNDP—incorporating tritium, ($^3$H) in very high specific activity. This is the work of a team in Cambridge, with the collaboration of the Radiochemical Centre, Amersham, Bucks., and Roche Products Ltd, Welwyn Garden City. It started in 1953 with the preparation of derivatives of MNDP and some related compounds, labelled with $^{14}C$, $^{82}Br$, $^{131}I$ and finally tritium, with progressively increasing specific activity (Maxwell, 1954, 1955; Marrian and Maxwell, 1956*a*, *b*; Marrian, 1957; Horwitz *et al.* (1959); Andrews *et al.*, 1962; Hodgson, 1968; Thomas, personal communication, 1970). The various aspects of this investigation and published work on other radioactive drugs have been reviewed (Mitchell, 1965, 1967, 1970, 1971*a*, *b*).

*Relevant properties of tritium and approximate clinical dosimetry*

Tritium ($^3H$) appears to be very suitable for incorporation into a radioactive drug. Its radiobiological action is localized in single cells or cell constituents. It is a pure beta ray emitter with very soft beta radiation which behaves biologically like a low LET radiation. The beta particles are completely absorbed in about 6 $\mu$m in water and soft tissue of unit density. The mean energy of the beta particles is $5{\cdot}73 \pm 0{\cdot}03$ keV and the corresponding range in unit density tissue is approximately 0·9 $\mu$m. The radioactive half-life is 12·4 years but almost invariably the values of the biological half-life (BHL) observed for human tissues and tumours with the radioactive drugs T-MNDP have been very much less, often between 7 and 14 days for the main half-life, though sometimes less, and in some cases greater for tumour than for normal tissues (Chipperfield, 1967*a*). Values of the main half-life found for carcinoma of the rectum were 9 and 13 days; a value of 3–4 days was observed in a case of squamous carcinoma of the skin.

It is useful to introduce the concept of the Differential Absorption Ratio (DAR) defined as the ratio of the specific activity of the tumour—or relevant normal tissue—to the mean specific activity of the body as a whole. An important step in the investigation of the individual patient is the measurement of the specific activity of tumour tissue, and also at least one normal tissue, if possible. There are, of course, some cases where it is not practicable to carry out such procedures.

The most important quantitative relationship concerning the dosimetry is that for uniformly distributed tritium at a stationary concentration, 1 mCi per g of tissue delivers a dose rate of 293 rad per day (24 h).

As a first approximation, calculations of radiation dose based on the assumption of uniform distribution of the tritium in normal tissues and tumours have proved useful.

Assuming uniform distribution throughout the body, 1 Ci of tritium administered in a single injection to a 70 kg patient delivers a dose of 18·1 rad for a BHL of 3 days, 36·2 rad for BHL 6 days, 54·4 rad for BHL 9 days, 72·5 rad for BHL 12 days and 78·5 rad for BHL 13 days (Mitchell *et al.*, 1963). The approximate values of the equivalent single doses, if the radiation were delivered at a high dose rate, would be for the different values of BHL, about 8, 14, 21, 27 and 29 rad respectively.

For most of the patients treated by intravenous T-MNDP, repeated injections have been given at intervals, often of 3 or 4 days, which in general are considerably less than the BHL in the tumour but are comparable with the BHL in the body as a whole, as shown by excretion studies. The approximate values of the doses delivered to normal tissues and tumours have been calculated, usually by means of a two-exponential model, with corresponding values of a short initial half-life and a longer main half-life (Chipperfield, 1967*a*). It was found that in most cases a power law gave a good or even better fit for the dependence of specific activity upon time but with this method calculation of the dose delivered was more difficult (White, 1973).

For doses calculated on the assumption of a uniform distribution of the tritium of T-MNDP in normal tissues and tumours, it appears that in most cases the clinical results are not inconsistent with experience of external radiotherapy, if the further *assumption* is made that the RBE has the value of 1·5 in relation to $^{60}$Co gamma radiation. Such a value is probably not inconsistent with previous experimental findings (see *e.g.*, Hall, Oliver and Bedford, 1967, and our own EM-autoradiographic studies, noting also the work of Cleaver, 1971).

However, the basis for these calculations can only be regarded as a crude first approximation. In general, in addition to macroscopic inhomogeneity there is inhomogeneity of the microscopic distribution of the tritium, which has been studied by morphometry and by autoradiography including EM-autoradiography, in addition to measurements of the specific activity of the tritium in macroscopic specimens. With regard to the human tumours studied (Chipperfield, 1967*a*), the initial uptake observed in biopsy and surgical specimens taken 30 min after intravenous or intra-arterial injection was highest in cases of carcinoma of the gastrointestinal tract, with values of 22·1 $\pm$ 8·4 $\mu$Ci per g wet weight per Ci injected for TRK 219 and 27·4 $\pm$ 2·2 $\mu$Ci per g per Ci injected for TRA 119. In some of these tumours and their metastases there was abundant stroma which showed low uptake, so that the mean specific activity of the tumour cells may be considerably higher than the average value for the macroscopic specimen, *e.g.*, in one case by a factor of 6·3. There is often wide variation among the tumour cells and in different parts of the tumour. Taking into account this distribution of activity, it is possible to calculate an approximate value of the *mean tumour cell* dose, but difficult to estimate the minimum tumour cell dose, even though the uptake is limited to viable tumour cells. In a typical example of carcinoma of the colon, the specific activity of the gross specimen may be 20 $\mu$Ci per g per Ci injected, so that allowing for stroma and necrotic areas by means of a factor of 3·5, the mean specific activity of the tumour cells would be 70 $\mu$Ci per g per Ci and for a single dose of 10 Ci injected 0·70 mCi per g; the corresponding initial mean tumour cell dose would be 205 rad per 24 h. For a typical value of the biological half-life of 10 days and mean life 14·4 days, the total mean cell dose delivered would be 2950, say of the order of 3000 rad. Assuming a value of the RBE of 1·5, the total mean tumour cell dose would be equivalent biologically to about 4500 rad of $\gamma$ radiation delivered continuously over about 14 days. Examples of calculations for the repeated small doses generally used in the treatment of individual patients will be discussed in considering the results.

From the point of view of detailed dosimetry, as a further extension of the ordinary (light microscopic) autoradiography (Mitchell *et al.*, 1963) quantitative EM-autoradiographic studies have been carried out on tumour cells in sections from patients treated with T-MNDP. Significant concentration, often by factors of 3–5, was found in the nucleoli and nucleolus associated chromatin, mainly in the mid and late S and early G2 phases.

It is to be noted that the introduction of tritium as a hydrogen isotope into an organic molecule such as MNDP causes no change in its chemical structure or pharmacological properties. Almost certainly there is no significant isotope effect. Moreover, the properties of tritium and of T-MNDP and its metabolism are such that the handling of these preparations presents no serious difficulties in therapeutic applications. The health and safety precautions described (Mitchell, 1965) have been extended to the treatment of outpatients with unit amounts of up to 5 Ci but usually not more than 3 Ci by intravenous injection.

### MATERIALS AND METHODS

*Preparations of T-MNDP used.*—The first preparations of T-MNDP, called T1, were made by the Wilzbach procedure but some of these appeared to contain tritiated water as an impurity, which was toxic to the bone marrow. The specific activity of T1 corresponded to approximately one atom of tritium per molecule of MNDP. These preparations were used from 4 February 1959 to 17 August 1960 for the treatment of cases Nos. 1–27.

Since September 1960 all the preparations of T-MNDP have been made by chemical synthesis, the tritium being incorporated in firmly bound positions by means of catalytic dehydrogenation of the corresponding halogen derivatives (Andrews *et al.*, 1962). We described these preparations following the catalogue of the Radiochemical Centre: TRA 72, containing theoretically one atom of tritium in the 6-position, had maximum specific activity 29·1 Ci/mmol, corresponding to 1 Ci in 14·64 mg of compound, and was used from 21 September 1960 to 5 May 1961 for the treatment of cases Nos. 28–48. TRA 119 contained up to 3 atoms of tritium per molecule in the 5, 6 and 7 positions, with maximum specific activity 87·3 Ci/mmol, corresponding to 1 Ci in 4·88 mg, and was used from 7 March 1962 to 12 December 1963 in the treatment of cases Nos. 49–99. A more reliable preparation was the next one, TRK 219, which contained 2 tritium atoms per molecule in the 6 and 7 positions with maximum specific activity 58·2 Ci/mmol, corresponding to 1 Ci in 7·32 mg, and was used from 24 September 1964 to 19 June 1970 for cases Nos. 100–224. Attempts were then made again to prepare the compound with 4 tritium atoms per molecule in the 5, 6, 7 and 8 positions (Hodgson, 1968; Thomas, personal communication, 1970). This preparation, TRK 397, varied in specific activity among 8 batches from 83 Ci/mmol up to 105·5 Ci/mmol, corresponding to 3·63 tritium atoms per molecule and 4·03 mg per Ci, and was used from 18 August 1970 to 12 September 1972 for cases Nos. 225–260. The preparation, TRQ 347, of specific activity 70 Ci/mmol was used from 6 February 1973 to 20 March 1973 for cases Nos. 261, 262 and 264. The preparation, TRQ 368, of specific activity 85 Ci/mmol was used from 1 May 1973 to 28 June 1973 for cases Nos. 263, 265 and 266. The solutions are sterilized by filtration into rubber capped bottles and frozen; the bottles of solution are stored in the gases above liquid nitrogen at about −196°C in a commercial "liquid nitrogen refrigerator" (Union Carbide Ltd). Storage at this temperature reduces the rate of decomposition by a factor of at least 5 (Evans, 1966).

With these radioactive drugs, one needs a high degree of radiopharmaceutical purity, as well as of radiochemical purity. Even now, the individual batches may differ in biological properties. For this reason every batch of T-MNDP is tested routinely on tissue cultures with autoradiography to be certain of a high degree of selective uptake in cells of a human tumour strain, Hep/2 (derived from a nasopharyngeal carcinoma) with negligible uptake into cells of normal origin, for which we now use mouse L cells. In earlier stages of the testing, other cells were included, *viz.*, HeLa cells and normal cells of human amniotic epithelium and embryonic skin, and lung epithelium, as well as monkey kidney cells (Simon-Reuss, 1961; Dendy, 1969; Morley and Dendy, 1973). A "bad" batch shows uptake into the cells of normal origin. Quantitatively, "good" batches show from 30 up to even 100 times the uptake into the tumour cells compared with that in the normal cells. These tests also provide a check for sterility. In addition, every batch has been tested to exclude acute toxicity—which has never been observed after intravenous injection in rabbits in doses corresponding to 11 Ci and 22 Ci/70 kg body weight. These rabbits have been kept for the rest of their life span for studies of possible late radiation effects.

*Pre-clinical testing.*—In this investigation, using the preparations of T-MNDP of very high specific activity, the chemical toxicity of MNDP is negligible (Mitchell, 1948, 1949). In addition to the tissue culture studies, many experiments have been carried out on mice, rats and rabbits; these have been continued throughout the years and included haematological studies on the rabbits. Di Vita (1964, personal communication) showed that in mice for a single intraperitoneal injection of TRA 72, the LD 50/30 days was about 20 mCi/g body weight, but only 1·1 mCi/g for tritiated water, for which there is essentially uniform distribution of the radioactivity; a value of about 1 mCi/g had also been found for tritiated water by Brues, Stroud and Rietz (1952). The results of our distribution studies with T-MNDP in rats with the Walker carcinosarcoma 256 were confirmed for mice with spontaneous mammary adenocarcinoma by Ganatra *et al.* (1969).

Information of great value from the point of view of the clinical applications in man was obtained in studies of the treatment of spontaneous malignant tumours and leukaemias in cats and dogs by our veterinary colleague, Dr I. A. Silver (Silver *et al.*, 1962). For example, 5 out of 7 cases of carcinoma of the tongue and palate in cats responded favourably to intra-arterial injection of quite small doses of T-MNDP and 2 of these lived longer than 2 years; tumours of these sites did not respond satisfactorily to external radiotherapy with 220 kVp x-rays. In addition, in long-term studies in 4 normal puppies aged 3 months (2 male and 2 female) injected with single doses of TRK 219 equivalent to 22 Ci/70 kg body weight, there was no effect on bone growth or epiphyseal closure. No abnormalities of any sort could be detected in any of the 4 in life or at post-mortem examination at 1, 2, 3 and 4 years respectively. The 2 longest surviving puppies, one male and one female, were mated with other dogs and fathered or produced normal litters. Subsequently they were mated together and also produced a normal litter. Measurements of the radioactivity of tissues taken at autopsy from other dogs showed relatively high activities in the eye (lens and retina), kidney, intestine and pancreas within a few hours of treatment. Nine months after treatment one case still had detectable activity in marrow and retina. No changes in the fundus of the eye or defects of vision were observed (Professor I. A. Silver, personal communication, 1970).

### *Clinical trials and relevant clinical investigations*

The design and analysis of the clinical investigations of T-MNDP have followed the general plan widely used for the examination of new chemotherapeutic agents (Brulé *et al.*, 1973). However, the problems involved were not as fully understood when these trials were started in February 1959 as at present and, moreover, there are essential differences between a radioactive drug such as T-MNDP and most chemotherapeutic agents. There is no serious problem of chemical toxicity because of the high specific activity of the preparations of T-MNDP used. There was already a substantial body of knowledge concerning the effects and hazards of internally administered radioactive isotopes, and very considerable clinical experience of the uses of $^{131}I$ and $^{32}P$ in inorganic form in therapy. The value and limitations of approximate dosimetry for these two isotopes had been emphasized in many investigations, including our own (Phillips, 1954, 1957; Phillips and Saunders, 1957; Phillips, Haybittle and Newberry, 1960; Mitchell, 1955, 1971*a*, *b*). With T-MNDP, the estimated radiation doses proved to be a reliable starting point as a first approximation, which enabled us to make rapid progress. Partly because of this, and also because no other basis would have been acceptable, we set out to include assessment of anti-tumour action in the Exploratory Phase, corresponding to Phase I, of our trials. Further, the investigations which can be regarded as within the scope of clinical pharmacology as applied to a radioactive drug have continued throughout most of Phase II; also the quality of the radioactive drug has improved steadily over the years.

In Phase I, we started the treatment of patients with "advanced or refractory tumours" using intra-arterial (i.a.) injection, mainly with the Seldinger technique as developed by Dr D. McC. Gregg (1958), and then used intravenous (i.v.) injections in "patients with generalized metastases" (Horwitz *et al.*, 1959). We noted that Gehan (1961) had shown that if a drug were effective in 20% of patients, or more, there would be a 95% chance of one or more successes in 14 consecutive cases. We found 5 "responses"—which were estimated on the modest basis of reduction in size of the tumour, or of part of the tumour—in the first 14 cases. Accordingly, we felt we must continue the investigation and we were encouraged by the relief of pain we observed in 5 of these patients.

From the beginning we started relevant investigations and first obtained evidence of uptake of T-MNDP in tumours after i.a. injection. Detailed studies of tritium uptake and distribution, half-lives and radiation doses, and excretion and metabolism have continued (Horwitz *et al.*, 1959; Marrian, Marshall and Mitchell, 1961; Marrian *et al.*, 1965; Chipperfield, 1967*a*, *b*; see also White, 1973). Summarizing our findings, including those on autopsies, after a single injection of 10 Ci of T-MNDP/70 kg body weight, the total doses, with standard deviation, received by normal tissues were estimated as follows: kidney $222 \pm 78$ rad, testis $180 \pm 29$ rad, bone marrow $83 \pm 65$ rad, liver $145 \pm 78$ rad, brain (long-lived component only) $137 \pm 46$ rad, skeletal muscle $54 \pm 26$ rad and small intestine $93 \pm 29$ rad.

Phase II, the main part of the trial so far, which is concerned with screening for clinical activity, has now been completed after the treatment of a further 200 cases. We selected the correct range of dosage from the beginning, so that the patients in both Phases I and II can be evaluated as a single group. All patients have been re-assessed in terms of present criteria. The selection of patients for treatment by means of T-MNDP was always discussed with colleagues. I examined all the patients myself, often throughout the treatment and at least at some stage of the treatment and follow-up; in most cases, I gave the intravenous injections myself.

From 4 February 1959 to 13 July 1973 we have treated, with doses of at least 1 Ci of the various preparations of T-MNDP, a total of 214 patients with different types of advanced and recurrent, inoperable, histologically verified malignant tumours and reticuloses. These tritiated drugs were used only when all other therapeutic measures—surgery, radiotherapy, chemotherapy and hormone therapy—had failed or were definitely contra-indicated. Many of the patients were very ill and in poor general condition.

*Assessment of the results of treatment with T-MNDP*

The following criteria were used for assessment:

*Curative.*—Well and free from clinical evidence of disease at 5 years or more after first treatment with the radioactive drug, noting whether alive or dead.

*Palliative.*—Survival after first treatment with the radioactive drug, and

*Clinical responses* (Hill and Larsen, 1972; Gerner and Moore, 1973) were defined as follows: *C.R.*—complete response. All measurable signs and all symptoms of tumour disappear; *P.R.*—partial response. At least a 50% decrease in the maximum cross-sectional area of the tumour without simultaneous progression of disease elsewhere; *Inc.R.*—incomplete response (extension of the term "lesser response" as defined by Gerner and Moore, 1973) including: (a) reduction of the size of the tumour with less than a 50% decrease in the maximum cross-sectional area, or (b) more than a 50% decrease in the maximum cross-sectional area of the tumour or of parts of the tumour, with simultaneous progression of the disease elsewhere, or (c) significant improvement of general condition and increase in performance status (Karnofsky *et al.*, 1951) or (d) useful relief of pain, if possible assessed in terms of reduction of the amounts of analgesic drugs required, or (e) relief of intractable pruritus, or more than one of these criteria.

Responses were required to persist for at least 4 weeks.

*None.*—No significant clinical response. This includes: no change or stationary disease, and progression, defined as a measurable increase in the maximum cross-sectional area of the tumour greater than 25%, or appearance of new lesions, or definite worsening of the patient's general condition.

*N.E.*—Non-evaluable treatments were studied, and classified as follows: Not evaluable because treatment was initiated prophylactically for poor prognosis as adjuvant therapy following, or in association with, radiation therapy or surgery, including potentially curative measures, or not curable by standard means, but no lesions were evaluable for measurement. Included were patients who were treated with radiation therapy and chemotherapy simultaneously or previously and in this case died within 1 month of the complications of the previous treatment.

### RESULTS

The results of treatment of the 162 patients with the various preparations of T-MNDP administered by intravenous injection are summarized in Table I. Of these patients, 151 were evaluable. The numbers of patients showing the different types of clinical response are listed and the footnotes give additional information.

Table I includes the total dose in Ci received by patients showing some clinical response. Except for single doses of up to 4 Ci, these total doses were delivered in a series of repeated injections, often 2–3 times weekly, each single injection usually not exceeding 3 Ci. Mention must be made of 2 patients (Cases Nos. 119 and 157) who received exceptionally

TABLE I.—*Summary of Results of Treatment with Tritiated MNDP Administered by Intravenous Injection*

Histologically verified cases

| Site and type of tumour | Total no. of patients | No. not evaluable | No. of patients evaluable | Clinical responses among all patients C.R. | P.R. | Inc.R. | None | Comments and total dose in Ci received by responders |
|---|---|---|---|---|---|---|---|---|
| Ca stomach | 19 | 2 | 17 | 0 | 0 | 0 | 17 | No responses |
| Ca colon | 33 | 2 | 31 | 0 | 1[a] | 3 | 27 | 11·2, 11·3[a], 19·6 and 58 Ci |
| Ca rectum | 13 | 3 | 10 | 0 | 0 | 3 | 7 | 5·7, 8·5 and 15·0 Ci |
| Ca pancreas | 7 | 0 | 7 | 0 | 2 | 1 | 4 | 15·4 and 22 Ci with P.R.; 9·0 Ci with Inc.R. |
| Ca breast[b] | 8 | 0 | 8 | 0 | 0 | 2 | 6 | 4 and 10·1 Ci |
| Ca ovary | 6 | 1 | 5 | 0 | 0 | 2 | 3 | 18·5 and 34 Ci |
| Malignant melanoma | 16 | 0 | 16 | 0 | 0 | 3 | 13 | 25, 37 and 58 Ci |
| Ca gall bladder and and bile ducts | 4 | 0 | 4 | 0 | 0 | 1 | 3 | 45 Ci |
| Sarcoma, various types | 12 | 0 | 12 | 0 | 0 | 0 | 12 | No responses |
| Ca bronchus | 10 | 2 | 8 | 0 | 0 | 0 | 8 | No responses |
| Hodgkin's disease | 3 | 0 | 3 | 1[c] | 0 | 2 | 0 | 10·0 Ci with C.R.[c]; 7·9 and 19·8 Ci with Inc.R. |
| Myelomatosis | 3 | 0 | 3 | 0 | 0 | 1 | 2 | 14·9 Ci |
| Other reticuloses and related malignancies | 6 | 0 | 6 | 0 | 0 | 1[d] | 5 | 3·1 Ci[d] |
| Malignant tumours of other sites, various types, mainly carcinoma | 22 | 1 | 21 | 0 | 0 | 0 | 21 | No responses |
| Totals | 162 | 11 | 151 | 1 | 3 | 19 | 128 | |

[a] Carcinoma became operable and was treated surgically 8 months after first injection; patient alive and well at 2 years after resection.
[b] Female patients; in addition one male patient showed no response.
[c] C.R. possibly curative: patient alive and well after 8 years with no evidence of disease.
[d] Chronic lymphatic leukaemia.

high total doses of 58 Ci given in 11 injections in 81 days and 19 injections in 154 days respectively; these patients will be discussed later.

No responses were observed in 17 evaluable patients in the trial with carcinoma of the stomach, 8 with carcinoma of the bronchus or the 12 with various types of sarcoma after intravenous injections of T-MNDP. Table II summarizes the results for the remaining 88 evaluable patients with sites and types of malignant diseases which showed some responses after treatment by means of intravenous injections. To overcome the problem that many of the patients were very ill and in poor general condition, in Table II separate consideration is given to the number and clinical responses of patients surviving 3 months or more after the first injection of the radioactive drug. In addition, the number of patients showing each type of incomplete response (Inc.R.) is shown.

Table I shows that among the 151 evaluable cases treated with intravenous injections, 23 (15·2%) showed some form of response, *i.e.*, C.R. (complete response), P.R. (partial response) and Inc.R. (incomplete responses). Table II shows that for the sites and types of malignant diseases which showed responses, some form of response was observed in 23 of the 88 patients, *i.e.*, 26·1%. Among the 42 patients surviving at least 3 months after the first intravenous injection there were, 1 C.R. possibly with a curative result in Hodgkin's disease, with recurrent

TABLE II.—*Summary of Results of Treatment with Tritiated MNDP Administered by Intravenous Injection*

Evaluable Histologically Verified Cases for Sites and Types of Malignant Diseases which Show Responses

| Site and type of tumour | No. of evaluable patients | Clinical responses among all evaluable patients | | | | No. and clinical responses of evaluable patients surviving 3 months or more after first injection of radioactive drug | | | | | No. of patients showing each type of Inc.R. | | | | |
|---|---|---|---|---|---|---|---|---|---|---|---|---|---|---|---|
| | | C.R. | P.R. | Inc.R. | None | No. | C.R. | P.R. | Inc.R. | None | a | b | c | d | e |
| Ca colon | 31 | 0 | 1[a] | 3 | 27 | 10 | 0 | 1[a] | 3 | 6 | 1 | 0 | 1 | 1 | 0 |
| Ca rectum | 10 | 0 | 0 | 3 | 7 | 9 | 0 | 0 | 3 | 6 | 0 | 0 | 1 | 2 | 0 |
| Ca pancreas | 7 | 0 | 2 | 1 | 4 | 5 | 0 | 2 | 1 | 2 | 0 | 0 | 0 | 1 | 0 |
| Ca breast[b] | 8 | 0 | 0 | 2 | 6 | 4 | 0 | 0 | 2 | 2 | 0 | 0 | 1 | 1 | 0 |
| Ca ovary | 5 | 0 | 0 | 2 | 3 | 3 | 0 | 0 | 2 | 1 | 0 | 0 | 2 | 0 | 0 |
| Malignant melanoma | 16 | 0 | 0 | 3 | 13 | 5 | 0 | 0 | 2 | 3 | 0 | 3 | 0 | 0 | 0 |
| Ca gall bladder and bile ducts | 4 | 0 | 0 | 1 | 3 | 1 | 0 | 0 | 1 | 0 | 1 | 0 | 0 | 0 | 0 |
| Hodgkin's disease | 3 | 1[c] | 0 | 2 | 0 | 3 | 1[c] | 0 | 2 | 0 | 1 | 0 | 0 | 0 | 1 |
| Myelomatosis | 3 | 0 | 0 | 1 | 2 | 1 | 0 | 0 | 1 | 0 | 0 | 0 | 0 | 1 | 0 |
| Chronic lymphatic leukaemia | 1 | 0 | 0 | 1 | 0 | 1 | 0 | 0 | 1 | 0 | 0 | 1 | 0 | 0 | 0 |
| Totals | 88 | 1 | 3 | 19 | 65 | 42 | 1 | 3 | 18 | 20 | 3 | 4 | 5 | 6 | 1 |

[a] Carcinoma became operable and was treated surgically 8 months after first injection; patient alive and well 2 years after resection.
[b] Female patients.
[c] C.R. Possibly curative; patient alive and well after 8 years with no evidence of disease.

cervical nodes after previous radiotherapy, 3 P.R., one of which was a case of carcinoma of the colon made operable and the other 2 of carcinoma of the pancreas, and 18 Inc.R., some of which were striking.

For intra-arterial administration, all 52 patients treated with doses of 1 Ci or more were evaluable. Table III summarizes the results and includes the total doses received by those patients showing some clinical response. Up to 3 intra-arterial injections were given in a number of these patients; the maximum dose given at one injection was 12 Ci. The numbers of patients in almost all the groups treated by intra-arterial injection are small; no responses were observed for carcinoma of the colon, rectum, ovary, bronchus or kidney and renal pelvis. Among 13 cases of inoperable cerebral glioma, there were no responses after intra-arterial therapy. In addition, in one case, No. 102, a man of 31, 3·8 Ci was injected into a cyst in an inoperable cerebral glioblastoma multiforme recurrent after previous external radiotherapy; the patient improved in general condition for at least 2 months [Inc.R.(C) (which could, of course, have been related to the aspiration)] and died at 11 months after the intracystic injection.

Table III shows that among the 52 patients treated by intra-arterial injection, 13 (25%) had some form of response. Table IV gives further details of the results of intra-arterial treatment with T-MNDP for the 18 patients with malignant diseases of sites and types which showed some responses. Among the 13 of these patients surviving at least 3 months after the first intra-arterial injection, there were 1 P.R., in a case of multiple metastases of malignant melanoma on the leg, with freedom from any evidence of disease for 3 years 4 months, and 9 Inc.R.

For all the evaluable patients in the trial treated by both the intravenous and intra-arterial routes, there was some form of response in 36 out of 203 (17·7%); however, for C.R. + P.R. there were only 6 responses out of 203 (approximately 3%). For the patients with sites and types of tumours which showed responses, some form of response was observed in 36

TABLE III.—*Summary of Results of Treatment with Tritiated MNDP Administered by Intra-arterial Injection*

Histologically Verified Cases, all Evaluable

| Site and type of tumour | Total no. of patients | Clinical responses among all patients: C.R. | P.R. | Inc.R. | None | Comments and total dose in Ci received by responders |
|---|---|---|---|---|---|---|
| Ca jejunum | 1 | 0 | 0 | 1 | 0 | 3 Ci |
| Ca colon | 3 | 0 | 0 | 1 | 2 | 4·2 Ci |
| Ca rectum | 5 | 0 | 0 | 0 | 5 | No responses |
| Tumours of kidney | 2 | 0 | 0 | 0 | 2 | No responses |
| Ca ovary | 3 | 0 | 0 | 0 | 3 | No responses |
| Seminoma testis | 1 | 0 | 1 | 0 | 0 | 3 Ci |
| Malignant melanoma | 4 | 0 | 1 | 2 | 1 | 16 Ci with P.R.; 2 and 25 Ci with Inc.R. |
| Cerebral tumours | 13[a] | 0 | 0 | 0 | 13 | No responses |
| Sarcoma, upper end of femur | 2[b] | 0 | 0 | 2 | 0 | 6 and 11 Ci |
| Ca bronchus | 4 | 0 | 0 | 0 | 4 | No responses |
| Ca cervix | 4 | 0 | 0 | 2 | 2 | 3·0 and 12·2 Ci |
| Ca vagina | 1 | 0 | 0 | 1 | 0 | 12 Ci |
| Ca antrum | 1 | 0 | 0 | 1 | 0 | 23·7 Ci |
| Ca parotid | 1 | 0 | 0 | 1 | 0 | 2·8 Ci |
| Malignant tumours of other sites, various types | 7 | 0 | 0 | 0 | 7 | No responses |
| Totals | 52 | 0 | 2 | 11 | 39 | |

[a] In addition, one case with Inc.R. after injection of 3·8 Ci into cyst.
[b] One fibrosarcoma and one chondrosarcoma.

TABLE IV.—*Summary of Results of Treatment with Tritiated MNDP Administered by Intra-arterial Injection*

Histologically Verified Cases, all Evaluable, for Sites and Types of Malignant Diseases which Show Responses

| Site and type of tumour | No. of patients, all evaluable | Clinical responses among all patients | | | | No. and clinical responses of patients surviving 3 months or more after first injection of radioactive drug | | | | | No. of patients showing each type of Inc. R. | | | |
|---|---|---|---|---|---|---|---|---|---|---|---|---|---|---|
| | | C.R. | P.R. | Inc.R. | None | No. | C.R. | P.R. | Inc.R. | None | a | b | c | d |
| Ca jejunum | 1 | 0 | 0 | 1 | 0 | 0 | 0 | 0 | 0 | 0 | 0 | 0 | 0 | 1 |
| Ca colon | 3 | 0 | 0 | 1 | 2 | 3 | 0 | 0 | 1 | 2 | 1 | 0 | 0 | 0 |
| Seminoma testis | 1 | 0 | 1 | 0 | 0 | 0 | 0 | 0 | 0 | 0 | 0 | 0 | 0 | 0 |
| Malignant melanoma | 4 | 0 | 1 | 2 | 1 | 3 | 0 | 1 | 2 | 0 | 0 | 1 | 0 | 1 |
| Sarcoma, upper end of femur | 2 | 0 | 0 | 2 | 0 | 2 | 0 | 0 | 2 | 0 | 2 | 0 | 0 | 0 |
| Ca cervix | 4 | 0 | 0 | 2 | 2 | 2 | 0 | 0 | 1 | 1 | 1 | 0 | 0 | 1 |
| Ca vagina | 1 | 0 | 0 | 1 | 0 | 1 | 0 | 0 | 1 | 0 | 1 | 0 | 0 | 0 |
| Ca antrum | 1 | 0 | 0 | 1 | 0 | 1 | 0 | 0 | 1 | 0 | 1 | 0 | 0 | 0 |
| Ca parotid | 1 | 0 | 0 | 1 | 0 | 1 | 0 | 0 | 1 | 0 | 0 | 0 | 0 | 1 |
| Totals | 18 | 0 | 2 | 11 | 5 | 13 | 0 | 1 | 9 | 3 | 6 | 1 | 0 | 4 |

out of 106 (34·0%); for C.R. + P.R. there were 6 among the 106 patients. Among the 55 of these patients surviving at least 3 months after the first injection, there was some form of response in 32 (58·2%); however, for C.R. + P.R. there were 5 out of the 55.

*Relevant details about some selected patients*

*Case No. 233* (Addenbrooke's Hospital No. 361274)

P.R. Female, 74 years. Carcinoma of descending colon high up near splenic flexure, inoperable, solidly adherent to the left kidney and other posterior abdominal wall structures. Moderately well-differentiated adenocarcinoma. On phenobarbitone for some years. Striking uptake was observed in scan with 6-$^{131}$I-iodo-MNDP. The total dose of 11·3 Ci of TRK 397 was given in 8 intravenous injections in 25 days. Body weight 57 kg. For 70 kg body weight, this is equivalent to 8 injections each 1·74 Ci, with total 13·9, say 14 Ci. Assuming a biological half-life (BHL) of 9 days and DAR = 5 for the tumour cells, the total mean dose delivered would be 3780 rad. With RBE = 1·5, the equivalent total mean dose of $^{60}$Co gamma radiation to the tumour cells is about 5700 rad delivered in a time probably equivalent to an overall time of 38 days, corresponding to the time between the first and last injections plus the assumed biological mean life of 13 days. There were no serious blood count changes. The minimum values reached were wbc 3000 and platelets 129,000/cm$^3$ at 29 days after the last injection.

A useful clinical response was obtained. The palpable mass disappeared from 2 to 7 months after the start of the injections but grew again slowly. The condition was then regarded as operable and was treated surgically at 8 months after the first injection, with " resection of splenic flexure with left kidney ". The patient was alive and well 2 years after the resection.

*Case No. 209* (Addenbrooke's Hospital No. 83494)

P.R. Male, 52 years. Carcinoma of tail of pancreas with metastases in liver—inoperable at laparotomy. Undifferentiated carcinoma with occasional acini, some containing mucus. Striking uptake was observed in scans with 6-$^{131}$I-iodo-MNDP. From measurements on a phantom model, the value of the macroscopic average DAR for uptake of 6-$^{131}$I-iodo-MNDP into the primary tumour mass was found to be 6–8 (say 7) (Mitchell, 1971*a*, p. 66 and Fig. 4). The total dose of 13·6 Ci of TRK 219 was given in 6 intravenous injections in 15 days. Body weight 54 kg. For 70 kg, this would be equivalent to 6 injections each of 2·93 Ci, with total 17·6 Ci. Sodium citrate mixture was given orally during the treatment to try to increase the uptake into the tumour. Assuming BHL 6 days and DAR = 7 for the tumour, the total mean dose delivered would be 4450 rad. With RBE = 1·5, the equivalent total mean dose would be about 6700 rad, delivered in an effective time of about 24 days. There were no serious blood count changes. The minimum values reached were wbc 2900 and platelets 195,000/cm$^3$ at 23 days after the last injection.

The abdominal mass below the left costal margin became only just palpable or not palpable for at least 4 weeks and then slowly enlarged. At 2 months after the end of the first course of injections, a second course was given to a total dose of 8·4 Ci in 3 injections in 7 days. There was only slight improvement in general condition, with a little reduction in size of the mass. The patient died 4 months 15 days after the start of the first course of injections.

*Case No. 266* (Addenbrooke's Hospital No. 339607)

P.R. Female, 23 years. Proven metastases liver and coeliac axis lymph nodes, almost in certainly primary carcinoma in body of pancreas—inoperable at laparotomy; also hypercalcaemia with no radiological evidence of metastases in bones. Poorly differentiated adenocarcinoma. The total dose of 15·4 Ci was given in 7 intravenous injections in 16 days. Body weight 41·5 kg. For 70 kg, this would be equivalent to 7 injections each of 3·71 Ci, with total 26 Ci. During the injections, the patient was treated with prednisone 30 mg daily and also chloroquine sulphate 200 mg tablets twice daily (as a DNA repair inhibitor; Mitchell, 1973); this was continued for 4 weeks after the injections. Assuming BHL 6 days and DAR = 5 in the tumour cells, the total mean dose delivered would be 4690 rad. With RBE = 1·5, the equivalent total mean dose would be about 7000 rad, delivered in an effective time of about 25 days. There were no serious blood count changes. The minimum values reached were wbc 3500 and platelets 103,000/cm$^3$ at 27 days after the last injection; the neutrophils showed toxic granulations.

There was a steady reduction in the size of the liver for at least 3 months and striking improvement of the general condition for at least 2 months. Then she deteriorated very slowly but finally died after about a week's illness with bronchopneumonia and pains in the back and legs, possibly due to spinal metastases, at 157 days after the first injection.

*Case No. 13* (Addenbrooke's Hospital No. 162632)

P.R. Male, 60 years. Massive recurrence in abdomen and pulmonary metastases from seminoma arising in undescended testicle, after surgery and large field radiotherapy, which resulted in a leucopenia reaching 1500 wbc/cm$^3$, and 2 blood transfusions. A single intra-aortic injection of 3 Ci of T1 was given. The catheter was inserted intra-arterially by the Seldinger method, with the tip in the aorta below the branch to the left subclavian artery. Body weight 58 kg. The abdominal mass shrank rapidly and became only just palpable. The oedema of the legs disappeared. The patient no longer needed morphia for the pain and he could lie flat in comfort. At 33 days after the injection rapid deterioration occurred, almost certainly due to a haemorrhage into tumour. The Hb fell to 8·1 g/100 ml and rapidly over 14 days the total wbc fell to 400 and the platelets from 115,000 to 10,000/cm$^3$. A blood transfusion was given but the patient died 9 days later. Autopsy showed complete necrosis of the para-aortic mass, hypoplastic sternal marrow and sheets of seminoma cells in the liver, lung parenchyma and lymph nodes adjacent to the left bronchus.

*Case No. 145* (Addenbrooke's Hospital No. 168033)

Inc.R.(c), Female, 57 years. Advanced recurrent carcinoma of ovary after previous incomplete surgery, radiotherapy and later chemotherapy with prednisone 15 mg daily orally and chlorambucil 10 mg daily which was tolerated for only 2 months. Mucinous cystadenocarcinoma. She was admitted to hospital "for terminal care" in very poor general condition (performance status 20–30%) with widespread metastases in the bones of the skull and pelvis, bilateral papilloedema, a subcutaneous mass over the left scapula and miliary carcinomatosis of the lungs, with bilateral pleural effusions containing malignant cells.

She was treated by a course of intravenous injections of TRK 219 to a total dose of 31·4 Ci in 10 injections in 34 days. Body weight 45·8 kg. The sternal marrow showed uptake in a group of cells regarded as tumour cells. Prednisone 10 mg daily was continued. (For body weight 70 kg, the total dose would be 48 Ci). Assuming BHL 6 days and DAR = 3, the total mean dose delivered would be about 5200 rad. With RBE = 1·5, the equivalent total mean dose would be about 7800 rad delivered in effective time about 43 days.

After being rather ill, though relieved by aspiration of the pleural effusion and a blood transfusion, she improved considerably in general condition, the palpable tumour masses became less tender and slightly smaller and the papilloedema decreased. She said that after the injections she felt like she did after the previous external radiotherapy.

The blood count showed serious changes at about 44 days after the first injection with minimum values Hb 7·3 g/100 ml, total wbc 1200, with lymphocytes 516 and platelet count 10,000/cm$^3$; rapid recovery occurred within 2 weeks after blood transfusions.

There was striking subjective improvement with P.S. 80–90% for 10 weeks. She acquired a new flat and new furniture and went away for a holiday. Then she deteriorated with increasing size of the tumour masses but with an essentially normal blood count. There was no response to a further single intravenous dose of 2·6 Ci of TRK 219. She died at 6 months 10 days after the first injection. Autopsy showed carcinomatosis. The bone marrow appeared normal; the kidneys were congested and showed some scarred glomeruli.

*Case No. 81* (Addenbrooke's Hospital No. 174744)

Inc.R.(e), Female, 20 years. Hodgkin's disease, probably of nodular sclerotic type, after previous radiotherapy and chemotherapy. On prednisone 20 mg daily orally. Dramatic relief of intractable pruritus about 4 h after a single intravenous injection of 2·9 Ci of TRA 119 in 21 mg of MNDP. Body weight about 67 kg. A further injection of 5 Ci was given 4 weeks later. There was no return of serious pruritus for the remaining 4 months of her life.

*Complications*

Haematological changes are the most obvious problem. A white blood cell count reduction to less than 2000 and/or platelet reduction to less than 25,000/cm$^3$ have been regarded as serious (Hill and Larsen, 1972). In previous untreated patients, doses of up to the equivalent of a total of 26 Ci given in 7 intravenous injections in 16 days/70 kg body weight resulted in no serious changes in blood count. The safety of a single injection of 11 Ci/70 kg body weight was confirmed for patients without serious bone marrow damage at a relatively early stage of this work (Mitchell *et al.*, 1963). The tolerance of the bone marrow can be greatly reduced by previous large field radiotherapy (see Case No. 13) and/or chemotherapy (see Case No. 115), and/or extensive involvement by growth. The maximum dose which could be given to a previously untreated patient without risk of serious bone marrow damage is probably about 45 Ci/70 kg body weight given in 10–15 injections in about 34 days.

Mention must be made of 2 patients who received exceptionally high total doses in probably unwise attempts to control advancing disease which had previously shown some response. Case No. 119, a man of 36 with advanced recurrent carcinoma of the colon and pulmonary metastases (body weight 66 kg) received a first course with a total dose of 40 Ci in 7 intravenous injections in 39 days; then after an interval of 31 days received a further 18·2 Ci in 4 injections in 11 days, but died 30 days later, the autopsy showing carcinomatosis and a hypoplastic bone marrow. Case No. 157, a man of 23 with recurrent generalized metastases of malignant melanoma, from a primary melanoma on the lower leg treated surgically (body weight 66 kg), received a total amount of 57·8 Ci in 19 intravenous injections approximately weekly in 154 days; from the second injection onwards, the intravenous injection was given in the hyperbaric oxygen tank at 3 atmospheres absolute pressure of oxygen, to try to increase the selective uptake into the tumour. At 12 weeks after the last injection, the Hb fell to 4·75 g/100 ml, the wbc to 2700 and the platelets to less than 10,000/cm$^3$. There were retinal haemorrhages, though it is to be noted that melanotic deposits were evident in each macular area about 8 weeks later. However, after blood transfusion the blood picture improved and the patient survived a further 14 weeks.

A clinical problem which has arisen is the occurrence of necrosis in tumour masses within the body after treatment with T-MNDP (see Case No. 13). This can lead to toxic absorption and a clinical picture similar to " crush syndrome "; this should probably be treated surgically if possible. A striking example was seen in a case (No. 125) of advanced carcinoma of the gall bladder with pulmonary metastases, treated with a striking immediate response, to a total dose of 45·2 Ci given in 8 injections in 99 days (body weight 48 kg). Toxic absorption from extensive necrotic tumour was probably the main cause of death at 25 days after the last injection; the autopsy showed almost aplastic marrow but the kidneys were undamaged.

In these trials there has never been evidence of renal damage. It is important to note that experience of the treatment of carcinoma of the thyroid with large doses of radio-iodine, $^{131}I$, has shown that there is no evidence of long-term damage to the kidney (Pochin, 1969, personal communication).

In one case (No. 52) of multiple metastases of malignant melanoma on the leg, intra-arterial therapy with TRA 119 reduced the tolerance of both skin and underlying bone to small field local radiotherapy; a normal dose of external 220 kVp x-rays resulted in a local radionecrosis, requiring surgical treatment and skin grafting.

Nausea is a not very common and never serious complication and has been experienced by some patients even after intravenous injections of 2–3 Ci; it is controlled almost invariably by oral perphenazine (Fentazin) 4 mg tablets; usually one tablet is taken about half an hour before the injection and another 2 h afterwards.

Three patients appeared to be made worse by intravenous injections of T-MNDP, one case (No. 97) with multiple recurrent secondary skin nodules of malignant melanoma, another (No. 68) also with multiple metastases of malignant melanoma and shortly afterwards developing cerebral metastases, and a case (No. 211) of advanced carcinoma of the colon which obstructed after the intravenous injection of 2·9 Ci, presumably due to local oedema. Another patient (Case No. 244) with an inoperable carcinoma of the colon, which had previously perforated and been treated surgically by palliative sigmoid colectomy, experienced abdominal pain even after single intravenous doses of 0·5 Ci of TRK 397; it was only possible to give a total dose of 2·5 Ci.

After intra-arterial injection, one case (No. 5) developed a femoral arterial mycotic aneurysm, 3–4 cm diameter, 3 months after catheterization as a result of wound sepsis.

*Ancillary procedures*

Many laboratory and clinical studies have been made to try to increase the selective uptake of T-MNDP into tumours. The most encouraging ancillary agent is prednisone, which appears to increase the alkaline phosphatase on the tumour cell surface in some cases; the effects on normal cells must be studied further. The possibilities of the use of phenobarbitone as an enzyme inducer should also be considered. Fasting appears to be of practical value (White, 1973). In *in vitro* experiments with Ehrlich ascites tumour cells it had been found that lack of glucose in the medium increased the dephosphorylation of MNDP but not of ATP (Fisher, 1968, personal communication; see Mitchell, 1971*a*, p. 46). This finding may explain the striking response, Inc.R.(b) in a patient (No. 103) with recurrent multiple metastases of malignant melanoma who also had diabetes, controlled on tolbutamide. The use of potassium citrate mixture to try to reduce the acidosis in tumours did not appear to be effective. Hyperbaric oxygen seemed to have some effect but it is doubtful whether this procedure is of sufficient practical value to justify its further study. Acetylcholine did not increase the uptake, as studied by scanning with 6-$^{131}$I-iodo-MNDP but produced local pain in the region of the tumour. It must be noted that tolazoline (Priscol) was used as a vasodilator with many of the intra-arterial injections. Recently, interesting results have been obtained by the use of chloroquine as a DNA repair inhibitor to try to increase the effects of the radiation delivered by the radioactive drug, perhaps selectively in the tumour cells (Mitchell, 1973).

We have made many attempts to select patients for treatment on the basis of measurements of the uptake of MNDP into the tumour, using the following investigations:

(i) Measurements of the specific activity of tritium in samples of tumour and normal tissue if possible. (It may be noted that a value of the DAR of 14 was found for a biopsy specimen of recurrent melanoma of the skin taken 35 min after intravenous injection of 0·5 Ci of TRK 397, but there was no clinical response to treatment.)

(ii) Autoradiographic studies on sections from operation and biopsy specimens in conjunction with measurements of the specific activity.

(iii) Autoradiographic studies of *in vitro* uptake by primary cultures of malignant cells freshly grown from specimens of tumour or ascitic fluid. Many of the cases for which the cells in culture showed uptake had been treated with prednisone.

(iv) Radioisotope scanning using the radioiodine labelled derivative, 6 - $^{131}$I - iodo - 2 - methyl - 1,4 - naphthaquinol - bis(di - ammonium phosphate), abbreviated 6-$^{131}$I-iodo MNDP. (Details of this work will be published elsewhere.) See Marrian *et al.* (1969).

### CONCLUSIONS

The results of the trials of T-MNDP as a radioactive drug in the treatment of selected patients with advanced malignant diseases of various types and sites are in general modest. The clinical results in some patients have been useful. It is likely that no responses can be expected for certain types and sites of tumour, *e.g.*, inoperable carcinoma of the stomach for treatment by intravenous injections, inoperable cerebral glioma for treatment by intra-arterial injections. It is concluded that much further investigation is desirable.

It is suggested that Phase III should be started, with controlled clinical trials, with proper design and random allocation of patients—perhaps with chemotherapy in the control groups—for the treatment of inoperable cases of carcinoma of the colon and of carcinoma of the pancreas.

Further studies of ancillary agents and procedures to improve the selection of patients appear to be necessary.

The possibilities of T-MNDP in the treatment of selected patients with advanced carcinoma of the rectum, breast and ovary and with advanced Hodgkin's disease and testicular seminoma have probably not been fully explored and appear to justify further investigation. Studies of rare tumours, such as primary hepatoma, should also be envisaged.

This work provides further evidence that tritium, when incorporated into a suitable compound, can produce therapeutic effects in patients with malignant diseases of certain sites and types. The scope and limitations of the effectiveness of T-MNDP as a radioactive drug appear to depend not only on the nature and properties of the carrier molecule, which are obviously of essential importance, but also on the biochemistry and pathological physiology of the tumour, especially on the blood supply and size of the tumour masses and the interrelationships of the tumour and the host.

I wish to acknowledge with grateful thanks the most helpful collaboration of the staff of the Radiochemical Centre, Amersham, and of Roche Products Ltd, Welwyn Garden City.

These investigations are the work of a team in Cambridge over many years. I wish to thank the many collaborators to whose work reference has been made, and many colleagues on the staff of the Radiotherapeutic Centre and the United Cambridge Hospitals. In addition, I wish to thank Mr E. A. King, Miss J. Young, Miss E. J. Porter and Dr J. L. Haybittle.

I wish to express my grateful thanks for financial assistance to the International Atomic Energy Agency, the Medical Research Council, the British Empire Cancer Campaign—now the Cancer Research Campaign—and my own Cancer Research Fund.

### REFERENCES

Andrews, K. J. M., Bultitude, F., Evans, E. A., Gronow, M., Lambert, R. W. & Marrian, D. H. (1962) A Radioactive Drug: 2-Methyl-6-Tritio-1,4-Naphthaquinol. Bis (Disodium Phosphate) and 2-Methyl-5,6,7-Tritritio-1,4-Naphthaquinol Bis (Disodium Phosphate). *J. chem. Soc.*, 3440.

Brues, A. M., Stroud, A. N. & Rietz, L. (1952) Toxicity of Tritium Oxide to Mice. *Proc. Soc. exp. Biol. Med.*, **79**, 174.

Brulé, G., Eckhardt, S. J., Hall, T. C. & Winkler, A. (1973) *Drug Therapy of Cancer.* Geneva: World Health Organization.

Chipperfield, B. (1967*a*) Tritium Uptake, Half-Lives and Radiation Doses in Tissues of Cancer Patients Treated with Tritiated 2-Methyl-1,4-Naphthaquinol Diphosphate. *Int. J. Cancer*, **2**, 381.

Chipperfield, B. (1967*b*) Excretion of Tritium after Therapeutic Injections of Tritiated 2-Methyl-1,4-Naphthaquinol Diphosphate in Cancer Patients. *Int. J. Cancer*, **2**, 396.

CLEAVER, J. E. (1971) Haematological Consequences of Radio-Isotope Incorporation, with particular reference to Tritium, Phosphorus and Strontium. In *Manual on Radiation Haematology*. Vienna: International Atomic Energy Agency.

DENDY, P. P. (1969) Selective Uptake of a Radioactive Drug into Human Tumour Cells Growing in Tissue Culture. *Acta radiol. ther. phys. biol.*, **8**, 514.

EVANS, E. A. (1966) Stability of a Radioactive Drug—Tetrasodium 2-Methyl-1,4-Naphthaquinol Diphosphate Labelled with Tritium. *Nature, Lond.*, **209**, 196.

GANATRA, R. D., RAMANATHAN, P., PATEL, M. C., MEHTA, M. N., SHAHANI, S. N., SHARMA, S. M., MANI, R. S., NORONHA, O. P. D. & BLAU, M. (1969) Radio-Iodinated Synkol as a Tumour-Localizing Agent. In *Medical Radioisotope Scintigraphy*, Vol. II. Vienna: International Atomic Energy Agency.

GEHAN, E. A. (1961) The Determination of the Number of Patients Required in a Preliminary and a Follow-up Trial of a New Chemotherapeutic Agent. *J. chron. Dis.*, **13**, 346.

GERNER, R. E. & MOORE, G. E. (1973) Multiple-Drug Therapy for Malignant Solid Tumors in Adults. *Cancer Chemother. Rep.*, Pt 1, **57**, 237.

GREGG, D. McC. (1958) Renal Aortography and Selective Renal Arteriography. *Postgrad. med. J.*, **34**, 149.

HALL, E. J., OLIVER, R. & BEDFORD, J. S. (1967) The Relative Biological Effectiveness of Tritium Beta Particles Compared to Gamma Radiation—Its Dependence on Dose-rate. *Br. J. Radiol.*, **40**, 704.

HILL, G. J. & LARSEN, R. R. (1972) Cancer Chemotherapy. I. Methods, Agents and Overall Results in 400 Patients. *Oncology*, **26**, 206.

HODGSON, A. (1968) Studies Related to the Synthesis of a Tritiated Derivative of 2-Methyl-1,4-Naphthoquinone. Ph.D. Dissertation, University of Cambridge.

HORWITZ, H., GREGG, D. McC., MARRIAN, D. H., MARSHALL, B. & MITCHELL, J. S. (1959) Clinical and Laboratory Studies of the Therapeutic Possibilities of Tritiated Synkavit, Mainly by Intra-arterial Administration. *Acta rad.* Suppl., **188**, 111.

KARNOFSKY, D. A., BURCHENAL, J. H., ARMISTEAD, G. C., SOUTHAM, C. M., BERNSTEIN, J. L., CRAVER, L. F. & RHOADS, C. P. (1951) Triethylene Melamine in the Treatment of Neoplastic Disease. *A.M.A. Archs internal Med.*, **87**, 477.

MARRIAN, D. H. & MAXWELL, D. R. (1956*a*) Tracer Studies of Potential Radiosensitising Agents: Tetrasodium 2-$^{14}$C-Methyl-1,4-Naphthohydroquinone Diphosphate. *Br. J. Cancer*, **10**, 575.

MARRIAN, D. H. & MAXWELL, D. R. (1956*b*) Tracer Studies of Potential Radiosensitising Agents: Tetrasodium 2-Methyl-3$^{82}$Br-1,4-Naphthohydroquinone Diphosphate and Tetrasodium 2,3-Dimethyl - 5,6 - di - $^{131}$I - iodo - 1,4 - benzohydroquinone Diphosphate. *Br. J. Cancer*, **10**, 739.

MARRIAN, D. H. (1957) Tritium-labelled 2-Methyl-1,4-Naphthoquinone and Confirmation of the Structure of its Adduct with Sodium Hydrogen Sulphite. *J. chem. Soc.*, 499.

MARRIAN, D. H., MARSHALL, B. & MITCHELL, J. S. (1961) Laboratory and Clinical Studies of Some Quinol Diphosphates and Related Compounds as Chemical Radiosensitizers and the Development of a Radioactive Drug. *Chemotherapia*, **3**, 225.

MARRIAN, D. H., MARSHALL, B., MITCHELL, J. S. & SIMON-REUSS, I. (1965) An Attempt to Develop a Radioactive Drug for Cancer Chemotherapy. In *The Treatment of Cancer*. Ed. J. S. Mitchell. Cambridge: University Press. p. 98.

MARRIAN, D. H., MITCHELL, J. S., BULL, C. H., King, E. A. & SZAZ, K. F. (1969) Labelled Compound Related to Synkavit and Its Uptake in Certain Human Tumours Studied by Radio-Isotope Scanning. *Acta radiol.*, **8**, 221.

MAXWELL, D. R. (1954) Some Experiments with Labelled Compounds Related to 2-Methyl-1,4-Naphthohydroquione Diphosphate (Synkavit). In *2nd Radioisotope Conference, Oxford*. London: Butterworth.

MAXWELL, D. R. (1955) Studies of Chemotherapeutic Agents, which Incorporate Radioactive Atoms and May Concentrate in Malignant Tumour Cells. Ph.D. Dissertation, University of Cambridge.

MITCHELL, J. S. (1948) Clinical Trials of Tetrasodium 2-Methyl-1,4-Naphthohydroquinone Diphosphate, in Conjunction with X-ray Therapy. *Br. J. Cancer*, **2**, 351.

MITCHELL, J. S. (1949) Histological Changes Produced by Large Doses of Tetra-sodium 2-Methyl-1,4-Naphthohydroquinone Diphosphate in Some Human Tumours. *Experienta*, **5**, 293.

MITCHELL, J. S. (1955) Isotopes. In *British Practice in Radiotherapy*. Ed.: E. Rock Carling, B. Windeyer and D. W. Smithers. London: Butterworths. p. 97.

MITCHELL, J. S., KING, E. A., MARRIAN, D. H. & CHIPPERFIELD, B. (1963) Investigation of a Radioactive Drug (TRA 119) With Special Reference to Autoradiographic and Related Studies. *Acta radiol. B. ther. phys. biol.*, **1**, 321.

MITCHELL, J. S. (1965) An Attempt to Develop a Radioactive Drug. *Clin. Radiol.*, **16**, 305.

MITCHELL, J. S. (1967) The Use of Tritium-Labelled Compounds. Chapter from *Modern Trends in Radiotherapy*. Ed.: T. J. Deeley and C. A. P. Wood. London: Butterworths.

MITCHELL, J. S. (1970) Über die Möglichkeit der Entwicklung radioaktiver Pharmaka. *Fortschr. Strahlensch. Strahlenschutz in Forschung und Praxis*, 10. Stuttgart: Georg Thieme.

MITCHELL, J. S. (1971*a*) *Cancer. If Curable, why not Cured?* Cambridge: W. Heffer and Sons.

MITCHELL, J. S. (1971*b*) Neuere Untersuchungen zur Frage der Therapeutischen Anwendungen von Tritium-Markierten Organischen Substanzen. *Atomkernenergie*, **18**, 109.

MITCHELL, J. S. (1973) Clinical Trials of Radiosensitizers and Fractionation. *Br. J. Radiol.*, **46**, 481.

MORLEY, T. G. & DENDY, P. P. (1973) Mechanisms of Selective Uptake of 2-Methyl-1,4-Naphthoquinol Bis Disodium Phosphate Into Some Mammalian Cells in Tissue Culture. *Br. J. Cancer*, **28**, 55.

PHILLIPS, A. F. (1954) The Gamma-ray Dose in Carcinoma of the Thyroid Treated by Radioiodine. *Acta radiol.*, **41**, 533.

PHILLIPS, A. F. (1957) Choice of a Radioactive Isotope for the Treatment of Carcinoma of the Thyroid. *Br. J. Radiol.*, **30**, 247.

PHILLIPS, A. F. & SAUNDERS, R. D. (1957) Autoradiography and Radioactivity Measurements with Human Neoplasms Containing Radiophosphorus. *Acta radiol.*, **48**, 101.

PHILLIPS, A. F., HAYBITTLE, J. L. & NEWBERY, G. R. (1960) Use of Iodine-124 for the Treatment of Carcinoma of the Thyroid. *Acta Un. int. Cancr.*, **16**, 1434.

SILVER, I. A., CATER, D. B., MARRIAN, D. H. & MARSHALL, B. (1962) Tritiated Tetra-sodium 2-Methyl-1,4-Naphthaquinol Diphosphate for Treatment of Spontaneous Tumours in Animals. *Acta radiol.*, **58**, 281.

SIMON-REUSS, I. (1961) $^{3}H$ Incorporation into Ascites Tumour and Tissue Culture Cells Exposed to Synkavit and its Tritiated Analogue. *Acta radiol.*, **56**, 49.

WHITE, C. (1973) Laboratory Studies of a Radioactive Drug Used in the Treatment of Patients with Cancer. Ph.D. Dissertation, University of Cambridge.